# Reduced H^+^ channel activity disrupts pH homeostasis and calcification in coccolithophores at low ocean pH

**DOI:** 10.1073/pnas.2118009119

**Published:** 2022-05-06

**Authors:** Dorothee M. Kottmeier, Abdesslam Chrachri, Gerald Langer, Katherine E. Helliwell, Glen L. Wheeler, Colin Brownlee

**Affiliations:** ^a^The Laboratory, Marine Biological Association, Plymouth PL1 2PB, United Kingdom;; ^b^MARUM Center for Marine Environmental Sciences, University of Bremen, 28334 Bremen, Germany;; ^c^Biosciences, College of Life and Environmental Sciences, University of Exeter, Exeter EX4 4QD, United Kingdom;; ^d^School of Ocean and Earth Science, University of Southampton, Southampton SO14 3ZH, United Kingdom

**Keywords:** coccolithophore, calcification, protons, channels, acidification

## Abstract

Coccolithophores are major producers of ocean biogenic calcite, but this process is predicted to be negatively affected by future ocean acidification scenarios. Since coccolithophores calcify intracellularly, the mechanisms through which changes in seawater carbonate chemistry affect calcification remain unclear. Here we show that voltage-gated H^+^ channels in the plasma membrane of *Coccolithus braarudii* serve to regulate pH and maintain calcification under normal conditions but have greatly reduced activity in cells acclimated to low pH. This disrupts intracellular pH homeostasis and impairs the ability of *C. braarudii* to remove H^+^ generated by the calcification process, leading to specific coccolith malformations. These coccolith malformations can be reproduced by pharmacological inhibition of H^+^ channels. Heavily calcified coccolithophore species such as *C. braarudii*, which make the major contribution to carbonate export to the deep ocean, have a large intracellular H^+^ load and are likely to be most vulnerable to future decreases in ocean pH.

## Significance

Coccolithophore calcification is a major ocean biogeochemical process. While this process is likely to be sensitive to acidification-driven changes in ocean carbonate chemistry, incomplete understanding of the underlying mechanisms and constraints is a major bottleneck in predicting ocean acidification effects on calcification. We report severe disruption of pH homeostasis linked to a loss of H^+^ channel function in the coccolithophore *Coccolithus braarudii* acclimated to seawater pH values that are likely to be encountered currently in localized regions and more widely in future oceans. This disruption leads to specific defects in coccolith morphology. These findings provide mechanistic insight into how calcification in different coccolithophores is affected by changes in seawater carbonate chemistry.

Anthropogenic CO_2_ emissions and the subsequent dissolution of CO_2_ in seawater have resulted in substantial changes in ocean carbonate chemistry (1). The resultant decrease in seawater pH, termed ocean acidification, is predicted to be particularly detrimental for calcifying organisms (2). Mean global surface ocean pH is currently around 8.2 but is predicted to fall as low as 7.7 by 2100 (3) and is likely to continue to fall further in the following centuries. Present-day marine organisms can experience significant fluctuations in seawater pH, particularly in coastal and upwelling regions (4, 5). Ocean acidification is therefore predicted to have an important influence not only on mean surface ocean pH but also on the extremes of pH experienced by marine organisms (6, 7).

Coccolithophores (Haptophyta) are a group of globally distributed unicellular phytoplankton that are characterized by their covering of intricately formed calcite scales (coccoliths). Coccolithophores account for a significant proportion of ocean productivity and are the main producers of biogenic calcite, making major contributions to global biogeochemical cycles, including the long-term export of both inorganic and organic carbon from the ocean photic zone to deep waters (8, 9). Unlike the vast majority of calcifying organisms, coccolithophore calcification occurs in an intracellular compartment, the Golgi-derived coccolith vesicle (CV), effectively isolating the calcification process from direct changes in seawater carbonate chemistry. Nevertheless, extensive laboratory observations indicate that ocean acidification may negatively impact coccolithophore calcification, albeit with significant variability of responses between species and strains (10–14). The negative impact on calcification rates occurs at calcite saturation states (Ω_calcite_) >1, indicating that it results primarily from impaired cellular production rather than dissolution (10, 15). However, prediction of how natural coccolithophore populations may respond to future changes in ocean pH are hampered by lack of mechanistic understanding of pH impacts at the cellular level (10).

As calcification occurs intracellularly using external HCO_3_^−^ as the primary dissolved inorganic carbon (DIC) source (16–18), coccolith formation is not directly dependent on external CO_3_^2−^ concentrations. However, the uptake of HCO_3_^−^ as a substrate for calcification results in the equimolar production of CaCO_3_ and H^+^ in the CV (18). In order to maintain saturation conditions for calcite formation, H^+^ produced by the calcification process must be rapidly removed from the CV, placing extraordinary demands for cellular pH regulation to prevent cellular acidosis (18).

Lower calcification rates under ocean acidification conditions appear to be primarily due to decreased pH rather than other aspects of carbonate chemistry (10, 19, 20). Coccolithophores exhibit highly unusual membrane physiology, including the presence of voltage-gated H^+^ channels in the plasma membrane (21) and a high sensitivity of cytosolic pH (pH_cyt_) to changes in external pH (pH_o_) (21, 22). Voltage-gated H^+^ channels are associated with rapid H^+^ efflux in a number of specialized animal cell types (23) and contribute to effective pH regulation in coccolithophores (21). As H^+^ channel function is dependent on the electrochemical gradient of H^+^ across the plasma membrane, this mechanism could be impaired under lower seawater pH. However, it remains unknown whether H^+^ channels play a direct role in removal of calcification-derived H^+^ or contribute to the sensitivity of coccolithophores to ocean acidification.

Coccolithophores exhibit significant diversity in their extent of calcification (*SI Appendix*, Fig. S1). The ratio of particulate inorganic carbon to particulate organic carbon (PIC/POC) of a coccolithophore culture is a measure of the relative rates of inorganic carbon fixation by calcification and organic carbon fixation by photosynthesis, respectively, and is commonly used as a simple metric to define the degree of calcification. The abundant bloom-forming species *Emiliania huxleyi* is moderately calcified (PIC/POC of around 1) and has been the focus of the vast majority of the studies into the effects of environmental change in coccolithophores (13). Coastal species belonging to the Pleurochrysidaceae and Hymenomonadaceae are lightly calcified, commonly exhibiting a PIC/POC of less than 0.5 (24–27). Species such as *Coccolithus braarudii*, *Calcidiscus leptoporus*, and *Helicosphaera carteri* exhibit much higher PIC/POC ratios and contribute the majority of carbonate export to the deep ocean in many areas (28–30). The physiological response of heavily calcified coccolithophores to ocean acidification is therefore of considerable biogeochemical significance. Growth and calcification rates in *C. leptoporus* and *C. braarudii* are sensitive to pH values predicted to prevail on a future decadal timescale (10, 15, 31, 32). However, a mechanistic understanding of the different sensitivity of coccolithophore species to changing ocean carbonate chemistry is lacking.

**Fig. 1.**
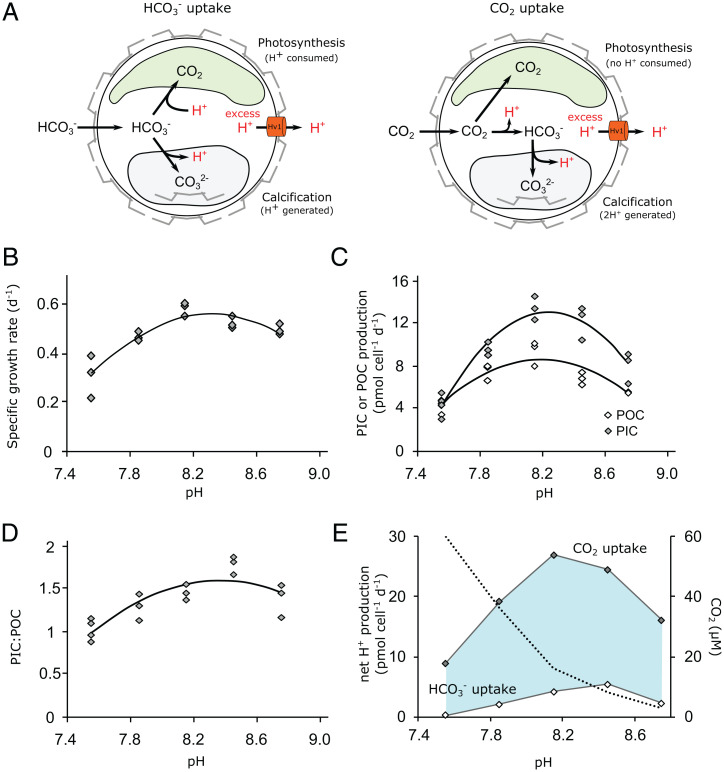
Physiology and H^+^ fluxes of *C. braarudii* cells grown at different seawater pH. (*A*) Schematic indicating H^+^ fluxes associated with photosynthesis and calcification in a coccolithophore cell. While many metabolic processes may contribute to the cellular H^+^ budget, these two processes are likely to be the major contributors. In a cell taking up HCO_3_^−^, the overall H^+^ budget is determined by the relative rates of H^+^ consumed during photosynthesis and H^+^ generated during calcification. In a cell taking up CO_2_, the H^+^ budget is determined primarily by calcification, as 2 H^+^ are produced for each molecule of CaCO_3_ produced and H^+^ are no longer consumed during photosynthesis. In both scenarios, excess H^+^ may be removed from the cell by H^+^ transporters in the plasma membrane, such as voltage-gated H^+^ channels (H_v_). Coccolithophores take up both HCO_3_^−^ and CO_2_ across the plasma membrane, with increasing proportions of DIC taken up as CO_2_ as seawater CO_2_ increases (34). (*B*) Growth rate of *C. braarudii* cells acclimated to different seawater pH. *n* = 3 replicates per treatment; line represents polynomial fit to mean. (*C*) Cellular production of POC through photosynthesis and PIC through calcification. The optima for both processes are close to the control conditions (pH 8.15). (*D*) As a consequence of the unequal changes in cellular POC and PIC production across the applied pH values, cellular PIC/POC ratios are minimal at pH 7.55 (∼1.0) and maximal at pH 8.45 (∼1.8). (*E*) Calculated net H^+^ budgets under the different pH regimes, based on rates of photosynthesis and calcification shown in *C* (see *Materials and Methods*). The concentration of CO_2_ in seawater is also shown (dashed line). Estimates are shown for cells using taking up only HCO_3_^−^ or only CO_2_. As *C. braarudii* cells will likely take up a mixture of both DIC species, with a shift toward greater CO_2_ usage at elevated CO_2_, the shaded area represents the potential range of H^+^ production. Regardless of DIC species used *C. braarudii* produces excess H^+^ at all applied pH values, but H^+^ production is much lower at pH 7.55 due to the decrease in calcification.

The net H^+^ load in a cell is determined by the combination of metabolic processes that consume or produce H^+^. H^+^ fluxes in coccolithophores will be primarily determined by the balance of H^+^ consumed by photosynthesis and H^+^ generated by calcification, with uptake of different carbon sources a particularly important consideration (Fig. 1*A*). CO_2_ uptake for photosynthesis results in no net production or consumption of H^+^, whereas uptake of HCO_3_^−^ requires the equimolar consumption of H^+^ in order to generate CO_2_. Growth at elevated CO_2_ causes a switch from HCO_3_^−^ uptake to predominately CO_2_ uptake in *E. huxleyi* (33, 34). The associated net decrease in H^+^ consumption will therefore increase the H^+^ load in coccolithophores grown at elevated CO_2_, which may exacerbate the potential for cytosolic acidosis caused by lower seawater pH.

In this study we set out to better understand the cellular mechanisms underlying the sensitivity of coccolithophore calcification to lower pH. We subjected the heavily calcified species *C. braarudii*, which is commonly found in temperate upwelling regions (35, 36), to conditions that reflect the range of pH values it may experience in current and future oceans. We show that acclimation to low pH leads to loss of H^+^ channel function and disruption of cellular pH regulation in *C. braarudii*. These effects are coincident with very specific defects in coccolith morphology that can be reproduced by direct inhibition of H^+^ channels. We conclude that H^+^ efflux through H^+^ channels is essential for maintaining both calcification rate and coccolith morphology. By providing a mechanistic insight into pH regulation during the calcification process, our results indicate that disruption of coccolithophore calcification in a future acidified ocean is likely to be most severe in heavily calcified species.

## Results

### Cellular H^+^ Load Varies with DIC Use for Calcification and Photosynthesis.

To examine more closely how the balance of photosynthesis and calcification may influence the cellular H^+^ load, we measured physiological parameters in *C. braarudii* cells acclimated to a broad range of carbonate chemistries (*SI Appendix*, Table S1). *C. braarudii* exhibited pH optima for growth and PIC and POC production of 8.32 ± 0.01, 8.20 ± 0.03, and 8.24 ± 0.02, respectively (pH_NBS_, *n* = 3, ± SE), with sharp declines in these parameters exhibited by cells grown at pH 7.85 and 7.55 (Fig. 1 *B*–*D*). PIC production decreased more strongly than POC production in acidified conditions, leading to lower PIC:POC ratios. These trends are in close agreement with other laboratory studies examining the response of *C. braarudii* to changing carbonate chemistries (10, 15, 31, 32, 37). Calculation of the H^+^ load from values of PIC and POC production indicated that H^+^ production by calcification exceeded H^+^ consumption by photosynthesis under all scenarios, being highest at optimal PIC:POC ratios (Fig. 1*E*). Although the large decrease in calcification rates at seawater pH 7.55 results in lower H^+^ production, the net H^+^ load could still be substantial due to a likely increase in CO_2_ uptake under these conditions (Fig. 1*E*) (34). The results illustrate that changes in the relative rates of photosynthesis and calcification, as well as in the carbon source used for photosynthesis, will have a major impact on the cellular H^+^ budget in *C. braarudii*, although in all cases there is a resultant requirement for net H^+^ efflux.

### Growth at Low pH Results in Specific Defects in Coccolith Morphology.

**Fig. 2.**
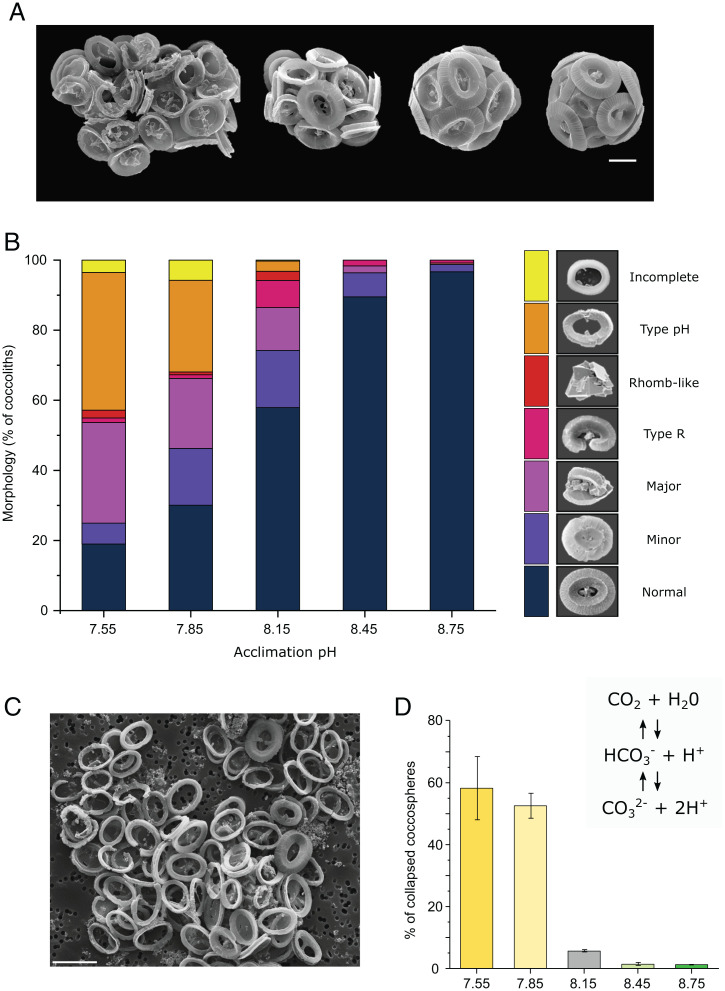
A unique defect in coccolith morphology occurs at low seawater pH. (*A*) Representative scanning electron micrographs of cells acclimated to (left to right) pH 7.55, 7.85, 8.15, and 8.75. The majority of cells grown at pH ≥8.15 had intact coccospheres without crystal or coccolith malformation. The majority of cells grown under acidified conditions produced malformed coccoliths, resulting in abnormal or collapsed coccospheres. (Scale bar, 5 μm.) (*B*) Quantification of coccolith malformations reveal an increasing proportion of defective coccoliths with seawater acidification. Coccoliths were grouped into morphological categories (see *Materials and Methods*). The morphological categories representing rhomb-like, R-type, major, and minor malformations are commonly observed *C. braarudii* cells grown under various stressors (76). However, the distinct “type-pH” was only observed in this study and appears unique to low-pH (high-CO_2_) conditions (*SI Appendix* Table S2). The counts represent the mean of three independent replicates. A minimum of 350 coccoliths were counted for each replicate. (*C*) An example of *C. braarudii* cells grown at pH 7.55 exhibiting a high proportion of the distinctive “type-pH” malformations. As the shield elements are not properly formed, the coccoliths are unable to interlock in the normal manner, resulting in the collapse of the coccospheres during preparation for SEM imaging. (Scale bar, 10 μm.) (*D*) The proportion of collapsed coccospheres increases at lower seawater pH. *n* = 3 replicates per treatment. Error bars represent SE. (*Inset*) The chemical equilibria of carbonate ions in aqueous systems.

Morphological defects in coccoliths are widely reported in coccolithophores grown under simulated ocean acidification conditions (37, 38), although there is little information on the specific nature of these malformations to aid mechanistic understanding of the impacts of low seawater pH on the calcification process. Scanning electron microscopy (SEM) analysis of coccolith morphology revealed that only 19.0 ± 5.0% and 30.1 ± 2.7% of coccoliths exhibited normal morphology at pH 7.55 and pH 7.85, respectively (*n* = 3, ± SE) (Fig. 2 *A* and *B*). Moreover, by performing a detailed categorization of each morphological defect, we found a very distinct “type-pH” malformation at low pH, in which the shield elements are malformed and greatly reduced in length so that the coccolith appears as a ring of calcite rather than a fully formed shield (Fig. 2*B*). This differs from an immature coccolith, in which the individual elements are reduced in length but correctly formed (*SI Appendix*, Fig. S2). Although the type-pH malformation has not been previously identified as a specific category of malformation, a similar phenotype can be observed in a previous study where *C. braarudii* was grown at low pH (37). Importantly, type-pH malformations are not observed under other stressors that cause extensive malformations, such as phosphate limitation or the Si analog, germanium (Ge) (39, 40) (*SI Appendix*, Table S2). Cells grown at low pH exhibited a large increase in the number of collapsed coccospheres observed by SEM analysis (Fig. 2 *C* and *D*), indicating that the extensive malformations result in an inability to maintain the structural integrity of the coccosphere. As the calcite saturation state (Ω_calcite_) was >1 in all scenarios, the defects in coccolith morphology are a consequence of impaired cellular production rather than dissolution.

The incidence of malformations in control cells grown at pH 8.15 can differ substantially between experiments (*SI Appendix*, Table S2). While many factors can influence the level of background malformations in laboratory cultures (41), our experiments indicate that culture pH must be tightly controlled to avoid pH-related effects on coccolith morphology. If they are allowed to grow to higher cell densities, coccolithophores can substantially lower culture media pH due to H^+^ generated from calcification. The control cultures (initial pH 8.15) reached a final pH of 8.01 (Fig. 2 and *SI Appendix* Table S1), which may have contributed to higher incidence of coccolith malformations in these samples.

### H^+^ Channel Function Is Greatly Reduced following Acclimation to Lower pH.

**Fig. 3.**
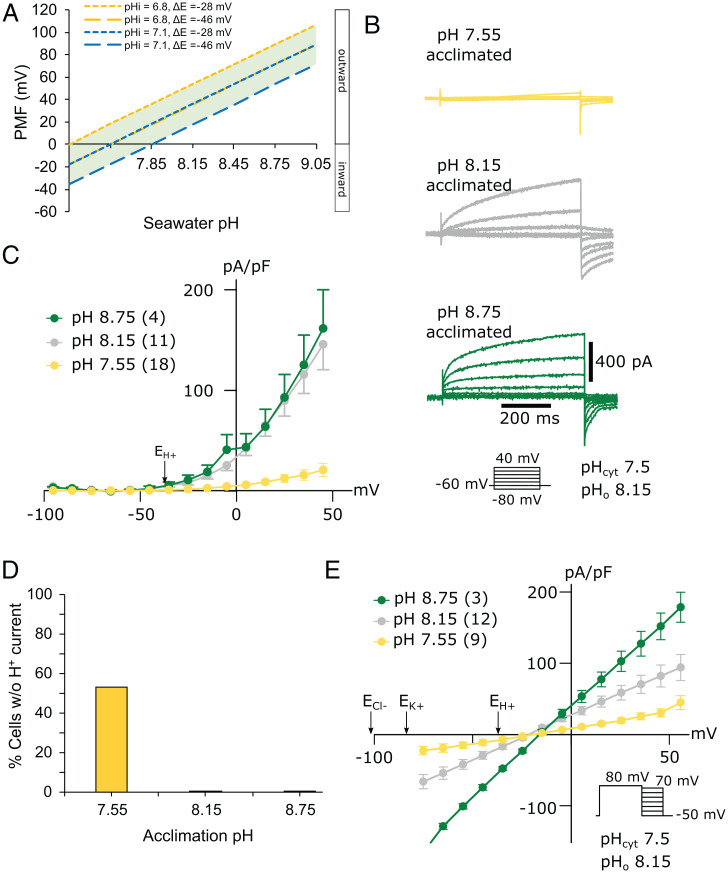
A reduced outward H^+^ current in *C. braarudii* cells acclimated to low seawater pH. (*A*) Estimation of the impact of changes in seawater pH on the pmf across the plasma membrane. Models of pmf based on measured maximal or minimal pH_cyt_ (pH 6.8 and 7.1) in combination with measured minimal and maximal ΔE [−46 mV and −28 mV (21)] suggest that pmf is close to zero at a seawater pH of approximately pH 7.55. Therefore, passive H^+^ efflux via voltage-gated H^+^ channels becomes unfavorable, unless mediated by excursions of pH_cyt_ (lower cytosolic pH) or V_m_ (depolarization). (*B*) Electrophysiological measurements of whole-cell currents in response to incremental 1-s 10-mV depolarizations from −80 to +40 mV performed in artificial seawater buffered to pH 8.15. The large outward-directed ion current is predominately carried by H^+^. The maximal current is much smaller in cells acclimated to pH 7.55. Example of currents from a single cell are shown for each pH treatment. For clarity only every other trace (Δ+20 mV) is indicated. (*C*) Mean whole-cell currents (plotted as pA/pF vs. mV) for acclimated cells. Outward currents are observed when the plasma membrane is depolarized to potentials more positive than the equilibrium potential of H^+^ (E_H+_; arrow). Cells acclimated to pH 7.55 exhibit a greatly reduced outward current. Values in parentheses represent *n*, and bars represent SE. (*D*) The proportion of cells that do not show an outward current (defined as an outward current <2.5 pA/pF at +45 mV) is greatly increased in cultures acclimated to a seawater pH of 7.55 compared to cultures acclimated to pH 8.15 or 8.75. *n* = 18. (*E*) Tail current analysis indicating that reversal potentials (E_rev_) are close to E_H+_ and more positive than the E_K+_ and E_Cl-_ in all treatments, suggesting that the observed currents in all treatments are predominately carried by H^+^. The reasons for the small deviation in reversal from theoretical E_H+_ are discussed in ref. 21.

To investigate how these defects in coccolith morphology could arise, we examined the physiology of *C. braarudii* cells grown at low pH. *C. braarudii* exhibits an unusual large outwardly rectifying H^+^ current at membrane potentials positive of the H^+^ equilibrium potential (E_H+_), due to the activity of voltage-gated H^+^ channels in the plasma membrane (21). Our previous studies showed that the H^+^ channel activation potential tracked E_H+_ cross the plasma membrane. At a resting membrane potential of −46 mV (21) there is a small net outward electrochemical gradient for H^+^ (proton motive force, pmf) across the *C. braarudii* plasma membrane at a seawater pH of 8.15 (Fig. 3*A*). The activation potential of the H^+^ current is close to the resting membrane potential, so any small positive excursion of membrane potential will activate the H^+^ current, leading to H^+^ efflux (21). H_v_ channels exhibit pH dependence of voltage gating (23). A decrease in pH_cyt_ acts to increase the outwardly directed pmf and causes a shift in the activation potential of the H^+^ current to more negative values, resulting in channel activation and net H^+^ efflux. The H^+^ efflux acts to hyperpolarize the membrane and increase pH_cyt_. These combined effects lead to the restoration of resting pH_cyt_ and inactivation of the H^+^ current (*SI Appendix*, Fig. S3 and *Supplementary Text*). The pH dependence of voltage gating means that H^+^ efflux through H^+^ channels is almost always outward (23). However, this characteristic also means that the operation of H^+^ channels is sensitive to changes in external pH. At pH_o_ 7.55 the activation potential shifts to more positive values, so channel-mediated H^+^ efflux would only occur following more substantial depolarization of the membrane potential and/or further reductions in pH_cyt_ (*SI Appendix*, Fig. S3).

In order to determine the impact of growth at unfavorable seawater pH on H^+^ channel function, we monitored H^+^ currents using patch-clamp recordings in *C*. *braarudii* cells previously acclimated to pH_o_ 7.55, 8.15, or 8.75. The mean amplitude of the outward H^+^ current, when measured at pH_o_ 8.15, was greatly reduced in cells that had been acclimated to pH 7.55 (Fig. 3 *B*–*D*); 52.9% of cells acclimated to pH 7.55 exhibited either greatly reduced or undetectable outward current (Fig. 3*D*), although these cells still displayed inward Cl^−^ currents typical of healthy *C. braarudii* cells (42) (*SI Appendix*, Fig. S4). The outward currents exhibited a reversal potential (E_rev_) close to E_H+_, indicating that H^+^ remained as the primary charge carrier in all cases (Fig. 3*E*). The results suggest that acclimation of *C. braarudii* to a low seawater pH unsuited to the operation of H^+^ channels results in the loss of H^+^ channel function.

### Calcifying Coccolithophores Possess Multiple H^+^ Channel Genes.

Homologs of the mammalian voltage-gated H^+^ channel, H_v_1, are present in coccolithophores and a range of other phytoplankton, although the large outward H^+^ currents typical of *C. braarudii* have not been reported in other algal cells, suggesting that H^+^ channels are utilized for alternative roles in noncalcifying cells [e.g., in supporting NADPH oxidase activity (43) or in dinoflagellate bioluminescence (44, 45)]. We previously characterized Hv1 channels from *E. huxleyi* and *C. braarudii* (21). Further analysis of haptophyte transcriptomes (46) revealed that coccolithophores possess an additional H^+^ channel homolog (Hv2) that was not found in noncalcifying haptophytes (*SI Appendix*, Fig. S5). The presence of this additional H_v_ homolog suggests that coccolithophore H^+^ channels may have undergone functional specialization related to calcification. In support of a specific role in calcification, we found that *HV1* and *HV2* were only expressed in the heavily calcified heterococcolith-bearing diploid life-cycle phase of *C. braarudii* and were not detected in the lightly calcified holococcolith-bearing haploid life-cycle phase (*SI Appendix*, Fig. S6). However, we did not find any significant change in the expression of either *HV1* or *HV2* in diploid cells acclimated to low pH (*SI Appendix*, Fig. S6). This suggests that the greatly reduced H^+^ conductance in these cells results from posttranscriptional or posttranslational regulation of H^+^ channels.

### pH Homeostasis Is Disrupted at Low Seawater pH.

**Fig. 4.**
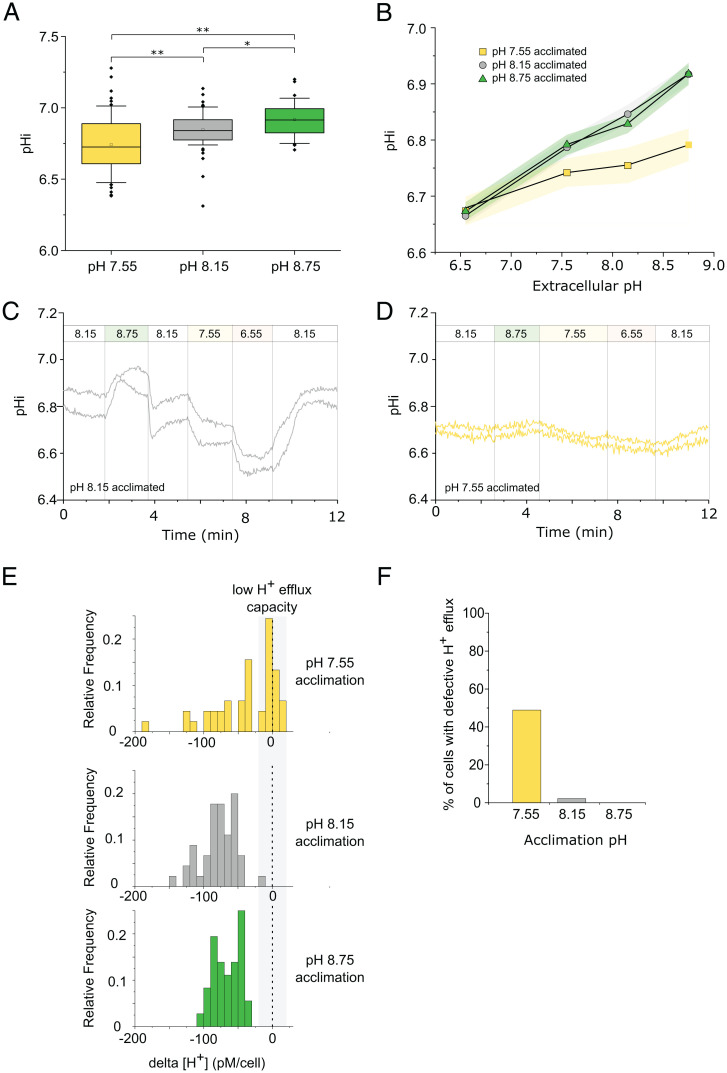
Changes in intracellular pH (pH_cyt_) in response to seawater acidification and alkalinization. (*A*) Intracellular pH (pH_cyt_) measured at acclimation pH conditions in *C. braarudii* cells loaded with the pH-responsive fluorescent dye SNARF-AM. Cells acclimated to pH 7.55 show a lower mean pH_cyt_ compared to those acclimated to pH 8.15 and pH 8.75. *n* = 63, 61, and 36 cells for pH 7.55, 8.15, and 8.75, respectively. One-way ANOVA, Holm–Sidak post hoc test, *P < 0.05, **P < 0.01. Box plots indicate mean (open square), median, 25th to 75th percentiles (box) and 10th to 90th percentiles (whiskers). (*B*) pH_cyt_ regulation following rapid changes in external pH. Acclimated cells were perfused with seawater at pH 6.55, 7.55, 8.15, and 8.75 for 2 min each to examine their ability to regulate pH_cyt_. Cells acclimated to pH 8.15 and 8.75 show the rapid adjustment of pH_cyt_ typical of coccolithophore cells (21, 22). However, cells acclimated to pH 7.55 show a much lower change in pH_cyt_ (*n* = 25, 61, and 36, respectively, for pH 7.55, 8.15, and 8.75). Shaded areas represent SE. (*C*) Example of rapid changes in intracellular pH (pH_cyt_) in cells acclimated to pH 8.15. Cells were perfused with ASW pH 8.15 and pH_cyt_ was monitored as the perfusion was switched to a higher or lower pH. Two representative cells are shown. (*D*) Example of cells acclimated to pH 7.55 exhibiting greatly decreased responsiveness in pH_cyt_ following changes in external pH. Note that the time course of the perfusion differs slightly from that shown in *C*. Two representative cells are shown. (*E*) Detailed examination of pH_cyt_ recovery during a transition from seawater pH 6.5 to seawater pH 8.15. The frequency histogram indicates the change in pH_cyt_ (shown as Δ[H^+^]) in individual cells acclimated to different pH regimes. While nearly all cells acclimated to pH 8.15 and 8.75 exhibit a substantial decrease in [H^+^] on transfer from pH 6.5 to higher pH, many cells acclimated to pH 7.55 are unable to respond, indicative of a defect in H^+^ efflux. *n* = 55, 61, and 36 cells. Cells acclimated to pH 7.55 exhibit a significantly different distribution to pH 8.15 or 8.75 (two-sample Kolmogorov–Smirnov test, *P* < 0.05). (*F*) Proportions of cells exhibiting defective H^+^ efflux in the experiment described in *E*. Defective H^+^ efflux was defined as a Δ[H^+^] less than 20 pM.

To determine whether the reduced H^+^ channel activity in cells acclimated to low pH led to disrupted cellular pH homeostasis, we examined resting pH_cyt_. The mean pH_cyt_ in cells acclimated to pH 8.15 was 6.85 ± 0.02 SE (*n* = 61). These pH_cyt_ values are similar to those estimated in *E. huxleyi* by multiple methodologies, suggesting that calcifying coccolithophores have a relatively low pH_cyt_ compared to other eukaryotes (47). Cells acclimated to pH 7.55 exhibited a significantly lower mean pH_cyt_ values than cells acclimated to pH 8.15 or pH 8.75 (Fig. 4*A* and *SI Appendix*, Fig. S7). Cells acclimated to pH 8.15 or 8.75 retained the ability to adjust intracellular pH rapidly within seconds when exposed to a higher or lower pH (21, 22), but this response was greatly reduced in cells acclimated to pH 7.55 (Fig. 4 *B*–*D*). To determine the capacity for H^+^ efflux, we transiently exposed cells to pH 6.5 and examined their ability to restore pH_cyt_ on transfer to pH 8.15. Nearly all cells acclimated to pH 8.15 or 8.75 showed a substantial decrease in cytosolic [H^+^] on transfer from pH 6.5 to 8.15 (Fig. 4*E*). However, many cells acclimated to pH 7.55 showed little or no capacity to lower cellular [H^+^] on transfer from pH 6.5 to 8.5, indicating the presence of distinct populations of responsive and unresponsive cells (Fig. 4 *E* and *F*). Thus, a significant proportion of cells acclimated to pH 7.55 exhibit a defect in H^+^ efflux, which likely reflects the highly reduced H^+^ channel activity in these cells (Fig. 3*D*). It should also be noted that cells acclimated to pH 7.55 are calcifying at a much lower rate than those acclimated to higher pH values (Fig. 1*C*), which will result in a much lower rates of intracellular H^+^ generation.

### Pharmacological Inhibition of H^+^ Channel Function Disrupts Coccolith Morphology.

**Fig. 5.**
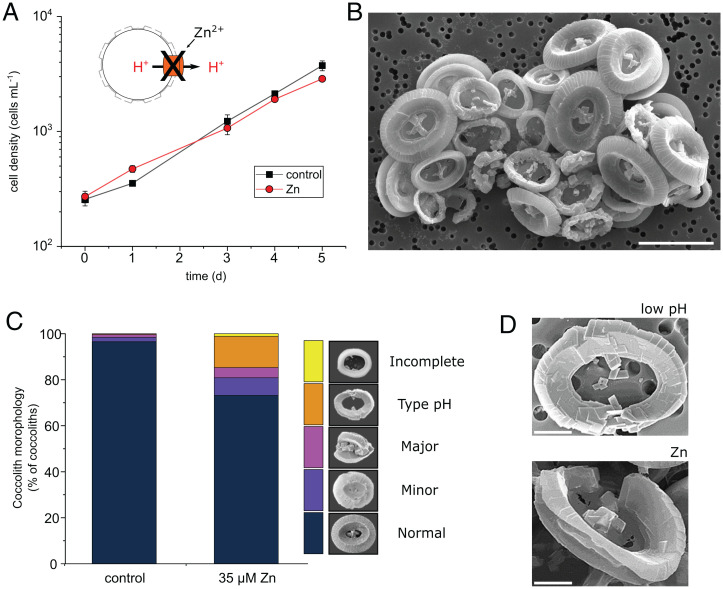
Effects of H_v_ inhibitors on coccolith morphology in *C. braarudii*. (*A*) Cell growth in the presence of the H^+^ channel inhibitor ZnCl_2_ (35 μM) at seawater pH of 8.15. *n* = 3. Error bars = SE. Application of a similar concentration of Zn (30 μM) results in a decrease of ∼50% in the amplitude of the outward H^+^ current (21). (*B*) SEM image of *C. braarudii* cells treated with ZnCl_2_ (35 μM) for 5 d showing the presence of many distinctive “type-pH” coccolith malformations. Note also the collapse of the coccospheres due to the inability of the coccoliths to interlock. (Scale bar, 10 μm.) (*C*) Quantitative analysis of coccolith morphology. Coccoliths were categorized into morphological categories (see *Materials and Methods*). The counts represent the mean of three independent replicate treatments. A minimum of 350 coccoliths were counted for each replicate. Cells exposed to 35 μM Zn exhibit a substantial increase in the proportion of the distinctive type-pH coccolith malformations. (*D*) Higher-resolution SEM images of type-pH morphological defects found in cells exposed to low pH (pH 7.55 from experiment described in Fig. 2) and Zn (35 μM). (Scale bar, 2 μm.).

Our results suggest that loss of H^+^ channel function and subsequent disruption of pH homeostasis is directly responsible for the defects in calcification exhibited by *C. braarudii* grown at low pH. To directly test this hypothesis, we treated cells with two inhibitors of H_v_ channels, Zn^2+^ (48) and 2-guanidinobenzimidazole (2-GBI) (49, 50). Cells grown in 35 μM Zn^2+^, which inhibits the outward H^+^ conductance in *C. braarudii* by ∼60% (21), showed only a small reduction in growth rate (control 0.54 ± 0.01 d^−1^ compared to Zn^2+^-treated 0.47 ± 0.01 d^−1^, *n* = 3, ± SE) (Fig. 5*A*). Moreover, treatment with Zn^2+^ did not affect other aspects of membrane physiology in *C. braarudii*, such as the inwardly rectifying Cl^−^ current (*SI Appendix*, Fig. S8). However, SEM examination of Zn^2+^-treated cells after 5 d revealed severe disruptions of coccolith morphology (Fig. 5 *B*–*D*). Importantly, Zn-treated cells exhibited the unique type-pH coccolith malformations, which were completely absent from control cells.

Micromolar concentrations of 2-GBI have very little immediate effect on H^+^ currents when applied extracellularly, because 2-GBI has limited membrane permeability and acts by binding to the intracellular side of the channel (49, 51). However, we found that prolonged exposure to 15 μM 2-GBI (>4 h) led to a substantial decrease in the outward H^+^ current in *C. braarudii* (*SI Appendix*, Fig. S9), suggesting that 2-GBI gradually becomes internalized in a manner observed for other guanidine derivatives (52). Other aspects of membrane physiology, such as fast action potentials (53) and inward Cl^−^ currents were not inhibited in these cells (*SI Appendix*, Figs. S8 and S9). Growth of cells in 15 μM 2-GBI for 5 d resulted in the presence of type-pH coccolith malformations (*SI Appendix*, Fig. S10), supporting the hypothesis that this calcification phenotype is specifically associated with impaired H^+^ channel function. Together, our results show that pharmacological inhibition of the H^+^ current leads to highly specific malformations in coccolith morphology (type-pH) that have only previously been observed in cells grown at low pH.

## Discussion

We have shown that voltage-gated H^+^ channels play a critical role in pH homeostasis during coccolith formation. H_v_ channels are regulated by the plasma membrane H^+^ electrochemical gradient and are primed to respond to decreases in intracellular pH, allowing rapid H^+^ efflux to restore intracellular pH (21). However, as extracellular pH decreases to values predicted in future ocean acidification scenarios, H^+^ efflux diminishes to the extent where this mechanism is no longer effective. Under such conditions, H^+^ channel function may only be maintained by reduced pH_cyt_ or depolarization of the membrane potential, with likely pronounced impacts on other physiological processes. Indeed, the loss of capacity to generate outward H^+^ currents shown here in cells acclimated to lower pH suggests a physiological need to switch off this mechanism of pH_cyt_ control after prolonged exposure to the lower pH. Our results also indicate that alternative mechanisms to maintain cytosolic pH homeostasis, such as energized forms of H^+^ transport (e.g., H^+^-ATPases, Na^+^/H^+^ exchangers), are incapable of dealing with the exceptional H^+^ load generated by intracellular calcification (*SI Appendix*, Table S3). Loss of H^+^ channel function will therefore lead to cytoplasmic acidosis unless calcification rate is reduced. The reliance on H^+^ channels for pH homeostasis may constrain the ability of coccolithophores to adapt to lower pH environments.

The inactivation of the outward H^+^ current due to prolonged exposure to low seawater pH most likely involves changes in protein translation or posttranslational modifications that modify channel activation. Elucidating these cellular mechanisms will be key in determining whether the loss of H^+^ channel function is reversible. Long-term experiments examining whether coccolithophores may eventually adapt to ocean acidification conditions have yielded complex results but support a trend of decreased calcification rates (54, 55). While physiological adaptations to low seawater pH are possible, such as recruiting alternative mechanisms for pH regulation or reducing calcification rates to lower the H^+^ load, these would either incur increased energetic costs or reduce the overall degree of calcification in future populations (*SI Appendix*, Table S3). The substantial heterogeneity in individual cell responses to low pH observed in the present study is also likely to be significant in determining how selection may operate on natural populations.

The sensitivity of *C. braarudii* to low pH is not only a consideration for future ocean scenarios but is also directly relevant to current oceans. *C. braarudii* is commonly found in temperate regions, including the Iberian and Benguela upwelling systems that are associated with significant variability in seawater pH (35, 36, 56). The lower pH extremes currently experienced within the Iberian upwelling system are within the range expected to cause coccolith malformations in our laboratory experiments (56). The ability of coccolithophores to cope with extremes of seawater pH may be critical in determining their response to a changing climate, as the frequency and severity of localized low pH events is predicted to increase dramatically in future oceans (57).

Calculated cellular H^+^ budgets differ considerably between coccolithophore species. In heavily calcified species, where calcification can occur at twice the rate of photosynthesis (10), rapid removal of excess H^+^ is essential. However, in lightly calcified species H^+^ production by calcification may be balanced by H^+^ consumption during photosynthesis, resulting in a much lower dependence on functional H^+^ channels. Calcification status may therefore be an important determinant in the sensitivity of coccolithophores to ocean acidification. A recent meta-analysis of multiple species revealed that the sensitivity of calcification rate to elevated seawater CO_2_ showed a strong positive correlation to PIC/POC ratio (11). Moreover, heavily calcified species such as *C. leptoporus* and *Gephyrocapsa oceanica* show highly malformed coccoliths under future ocean acidification conditions, whereas coccolith morphology in lightly calcified species, such as *Syracosphaera pulchra*, *Chrysotila carterae*, and *Ochrosphaera neopolitana*, is less sensitive (15, 25, 27, 58, 59). Indeed, evidence from boron isotope approaches indicated that *O. neopolitana* is able to maintain a constant pH in the CV over a range of seawater carbonate chemistries, although the pH range examined was relatively narrow (pH 8.05 to 8.35) (25). Our data provide mechanistic insight into the differential sensitivity of coccolithophore species, suggesting that H^+^ load is likely to be the key determinant of their sensitivity to ocean acidification. This conclusion is seemingly at odds with observations of overcalcified morphotypes of *E. huxleyi* at higher CO_2_ levels in the Southern Ocean (60) and millennial-scale trends indicating a correlation between increasing prevalence of “overcalcified” morphotypes of *E. huxleyi* with increased atmospheric CO_2_ over approximately the past 150,000 y (61). However, laboratory analyses of “overcalcified” *E. huxleyi* morphotypes suggest that this phenotype relates primarily to coccolith morphology rather than calcification rate, as their PIC/POC ratios are not higher than those with normal coccolith morphology (62). The overcalcified morphotype is present in cells grown at standard carbonate chemistries (e.g., 400 µatm CO_2_) (62), so it is not a specific indicator of low pH stress.

While nonspecific defects in coccolith morphology reflecting reduced calcification in response to ocean acidification have been observed in many studies (15, 37), the unique malformations observed here in *C. braarudii* now provide a mechanistic link between seawater pH, the ability to regulate pH_cyt_, and coccolith morphology. The highly specific nature of the *C. braarudii* malformations may facilitate the identification of low-pH stress in environmental populations and aid the characterization of past ocean acidification events in the fossil record (37). Modeled reconstructions indicate that, apart from the last *ca*. 25 My, surface ocean pH has been lower than at present over much of the 200 My since the emergence of calcifying coccolithophores (63, 64). This suggests that coccolithophores possess some capacity to adapt to the lowering of seawater pH over geological timescales. However, the very rapid predicted decline in surface ocean pH driven by anthropogenic CO_2_ emissions may limit the degree to which coccolithophores can adapt their physiology. Recent evidence indicates that the mass extinction event at the (K–Pg) boundary (66 Ma), which led to the loss of 90% of coccolithophore species, was associated with rapid ocean acidification (65). It is notable that many of the coccolithophore species that survived the K–Pg Cretaceous–Paleogene mass extinction event were coastal species (66–68), which may have been better suited to variable seawater pH and therefore less sensitive to ocean acidification.

Multiple environmental parameters in addition to carbonate chemistry are predicted to change in future oceans, including temperature, nutrient availability, and ecosystem-scale changes in the abundance of predators, pathogens, and competitors (69). Predicting the response of coccolithophore populations to future environmental change is therefore highly complex. Our incomplete understanding of the haplo-diplontic life cycle of coccolithophores further limits our ability to predict how natural populations may respond to unfavorable conditions (70). However, our results show that the physiology of heavily calcified species such as *C. braarudii* is best suited to a constant seawater pH and that calcification is likely to be severely affected by ocean acidification. The ability of coccolithophores to calcify intracellularly, which has facilitated the evolution of remarkably diverse coccolith architecture, required the development of specialized physiological mechanisms for pH homeostasis that ultimately may constrain the ability of certain species to adapt to rapid changes in ocean pH.

## Materials and Methods

### Cell Culturing.

Cultures of *C. braarudii* (PLY182g) (formerly *Coccolithus pelagicus* ssp. *braarudii*) were grown in sterile-filtered seawater containing additions of nitrate, phosphate, trace metals, and vitamins according to standard F/2 medium as described previously (42). Silicon, selenium, and nickel were also supplemented in concentrations of 10 µM, 0.0025 µM, and 0.0022 µM, respectively. Dilute-batch cultures were maintained at 15 °C under an irradiance of 50 µmol⋅m^−2^⋅s^−1^ with a 16:8-h light:dark cycle. Cells were cultured in in autoclaved borosilicate bottles with minimal headspace and gas-tight lids to avoid in- and outgassing of CO_2_ (Duran Group).

### Acclimation to Various Seawater pH.

Cultures were preacclimated for 4 d in a range of seawater pH/carbonate chemistry conditions and then used to inoculate test cultures (*SI Appendix*, Table S1). Triplicate cultures were used for all analyses, except for the pH_cyt_ measurements where five replicate cultures were grown. Growth rates and coccolith morphology were determined after 5 d in test conditions (i.e., a minimum of 9 d total acclimation, equating to approximately seven to eight generations for a specific growth rate between 0.45 and 0.6 d^−1^) (31). As coccolith secretion involves fusion of the CV to the plasma membrane, *C. braarudii* will exhibit significant rates of membrane cycling (71). Physiological measurements (pH_cyt_, patch clamp) were performed between 5 to 10 d after inoculation into test conditions. Adjustment of seawater pH/carbonate chemistry was performed by modulating total alkalinity (TA) with amounts of HCl or NaOH at constant DIC in sealed containers. Cell density was kept between 500 and 4,000 cells⋅mL^−1^ to minimize carbonate chemistry drifts. Carbonate chemistry was measured immediately after cell inoculation and at the end of the acclimation period measuring pH_NBS_ and TA with a pH meter (Mettler Toledo) and alkalinity titrator (TitroLine alpha plus; Schott Instruments), respectively. TA measurements were corrected with certified reference materials provided by A. Dickson, Scripps Institution of Oceanography, La Jolla, CA. Data were accuracy-corrected with certified reference materials supplied by A. Dickson. Calculations were made with CO2SYS (72).

### Phenotypic Changes in Physiology.

Cell growth was assessed by daily cell counting with Sedgewick Rafter counting chamber (Graticule Optics) using a Leica DM 1000 light-emitting diode light microscope. Specific growth rates (μ) were calculated from daily increments in cell concentrations counted every 24 or 48 h (73). Cellular POC content was estimated by measuring the area of decalcified cells microscopically. The area was converted to volume, assuming cells were spherical. The mean volume [cubic micrometers] of at least 20 to 50 cells per culture was converted into POC quota using the equation

$$\text{POC}\left[{\text{pg}\;\text{cell}}^{-1}\right]=\text{a}\times {\text{V}}^{\text{b}},$$

where a and b are constants (0.216 and 0.939, respectively) for nondiatom protists (74). The cellular PIC contents were also estimated microscopically, using the volume of the coccosphere. To obtain the cellular PIC quota, the volume of the coccolith is required. The following equation was used:

$$\text{Vc}=\text{ks}\times {\text{L}}^{3}.$$

Here, Vc is the volume of coccoliths and can be estimated using coccolith length L and the shape constant ks (75), which is 0.06 for *C. braarudii.* The cellular PIC quota is calculated from the following equation:

$$\text{PIC}\left[{\text{pg}\;\text{cell}}^{-1}\right]=\text{n}\times \text{Vc}\times \rho \times \left(\text{Mc}/{\text{Mcaco}}_{3}\right),$$

where n is the total number of coccoliths per cell including the discarded coccoliths, *ρ* is the calcite density of 2.7 pg⋅µm^−3^ assuming coccoliths are pure calcite, and Mc/Mcaco_3_ is the molar mass ratio of C and CaCO_3_.

Time points for sampling cell volumes (t = 10 h after the onset of the light phase) were chosen in order to present daily means (according to a modified version of the model provided in ref. 73). Production rates of POC and PIC (picomoles per cell per day) were approximated as cellular POC content [picomoles per cell] × μ × [day^−1^] or cellular PIC content [picomoles per cell] × μ × [day^−1^], respectively. Determination of pH optima was performed by determining the vertex of a polynomial fit (second order) of three independent experiments (each experiment consists of triplicate cultures acclimated to the five different seawater pH).

### Calculation of the pmf.

The pmf at the different seawater pH was estimated as

$$\text{pmf}=-2.30\text{3RT}/F\;\Delta \text{pH}+{\text{V}}_{\text{m}},$$

where *z* is the electrical charge of H^+^, *F* is the Faraday constant [joules per volt per mole], V_m_ is resting membrane potential [volts], R is the gas constant [joules per mole per Kelvin], and T is temperature [Kelvin]. We used a value of −46 mV for V_m_, which represents a mean of previously measured values in *C. braarudii* (21). To show how changes in pH_cyt_ and V_m_ may influence pmf during changes in pH_o_, we calculated pmf using two values for pH_cyt_ (6.8 and 7.1) and resting V_m_ (−46 and −28 mV).

### Calculation of H^+^ Production Rates.

H^+^ production and consumption during photosynthesis and calcification were calculated based on POC and PIC production rates. To determine the possible range for net H^+^ load, we estimated maximal and minimal values based on a cell taking up only CO_2_ (no H^+^ consumed per C fixed during photosynthesis, 2 H^+^ generated per C precipitated during calcification) or taking up only HCO_3_^−^ (1 H^+^ consumed per C fixed during photosynthesis, 1 H^+^ generated per C precipitated during calcification).

### SEM Analysis of Coccolith Morphology.

Samples for SEM analysis were filtered on polycarbonate filters (0.8-μm pore size), dried in a drying cabinet at 50 °C for 24 h, then sputter‐coated with gold‐palladium using an Emitech K550 sputter coater at the Plymouth Electron Microscopy Centre (PEMC). Scanning electron micrographs were produced with both Jeol JSM-6610LV and Jeol JSM-7001F at PEMC. The following categories were used to describe coccolith morphology of *C. braarudii*: normal; minor malformation (element malformation that does not impair interlocking); major malformation (shield malformation that impairs interlocking); malformation type-R (gap in both shields so that the shield elements do not form a closed oval shape); rhomb-like malformation (elements severely malformed displaying rhomb-like crystal morphology); incomplete (closed oval shape, but with incompletely grown shield elements that do not exhibit malformations); and type-pH (closed oval shape, but with short shield elements exhibiting malformations) (Fig. 2) (76). Despite a superficial similarity between the categories “incomplete” and “type-pH” that can make them difficult to distinguish in the light microscope, they are easily distinguished in SEM. An incomplete coccolith indicates that coccolith growth was stopped prematurely, but it does not indicate a malfunctioning of the morphogenetic machinery. Therefore, the label “incomplete” should only be applied to coccoliths that do not feature any malformations (41, 77). A malformed coccolith contains individual elements that have a disrupted morphology, rather than just an abnormal size. An average of ∼350 coccoliths were analyzed per replicate culture flask, with triplicate cultures examined for each treatment (78). Coccolith categorization and counting employed standard methodologies as described in detail in ref. 41.

### *C. braarudii* Patch-Clamp Recording and Analysis.

*C. braarudii* cells were decalcified by washing cells with Ca^2+^-free artificial sea water (ASW) containing 25 mM ethyleneglycol-*bis*(β-aminoethyl)-*N*,*N*,*N'*,*N'*-tetraacetic acid (21, 42). The recording chamber volume was 1.5 cm^3^, and solutions were exchanged using gravity-fed input and suction output at a rate of 5 cm^3^⋅min^−1^. Patch electrodes were pulled from filamented borosilicate glass (1.5-mm outer diameter, 0.86-mm inner diameter) using a P-97 puller (Sutter Instruments) to resistances of 3 to 6 MΩ. All external and pipette solutions are described in *SI Appendix*, Table S4. Sorbitol was added to pipette solutions to adjust the osmolarity to 1,200 mOsmol⋅kg^−1^. Liquid junction potentials were calculated using the junction potential tool in Clampex (Molecular Devices) and corrected off-line. Whole-cell capacitance and seal resistance (leak) were periodically monitored during experiments by applying a <5-mV test pulse. Currents were linear-leak-subtracted in Clampfit (Molecular Devices) using the pretest seal resistance. Current voltage relations were determined on leak subtracted families by measuring the maximum current observed after a 500-ms pulse. Reversal potentials were determined by manually measuring the peak tail currents of leak subtracted families of traces and calculating a linear regression versus test voltage (21). Series resistance was monitored throughout the experiments and whole cell currents were analyzed only from recordings in which series resistance varied by less than 15%.

### Application of H^+^ Channel Inhibitors.

ZnCl_2_ or 2-GBI was added to *C. braarudii* cultures to give a final concentration of 30 μM or 15 μM, respectively. Cell density was monitored daily to examine impacts of the inhibitors on growth, before cells were harvested on day 5 for SEM analysis of coccolith morphology. Note that extracellular pH plays an important role in the efficacy of Zn as a H^+^ channel inhibitor, as H^+^ competes with Zn^2+^ for the binding sites (79). In addition, the solubility of Zn^2+^ is limited in alkaline solutions due to the formation of Zn(OH)_2_ (80).

### Intracellular pH Measurements.

For pH_cyt_ measurements, *C. braarudii* cells were loaded with the cell-permeant acetoxymethyl ester of the pH-sensitive fluorescent dye carboxy SNARF-1. Cells were incubated with SNARF-1 (5 μM) for 20 to 40 min, before being washed with ASW and placed in a polylysine-coated imaging dish. Cells were imaged using a Nikon Ti Eclipse fluorescence (total internal reflection fluorescence) system, equipped with a Photometrics Evolve electron-multiplied charge-coupled device camera and a Photometrics DV2 beamsplitter. SNARF-1 was excited between 540 and 560 nm and fluorescence emission was captured at 580 nm (570 to 590 nm) and 630 nm (620 to 640 nm). Cells were inspected to ensure even cytoplasmic loading of SNARF-1. Cells showing very low fluorescence due to poor loading or very bright fluorescence relative to the average population were discarded from the analysis. Images were recorded at the rate of 3.3 frames per s (300-ms exposure). Background fluorescence was minimal and was therefore not subtracted.

pH_cyt_ values at acclimation conditions were derived by measuring SNARF-1 fluorescence in ASW with carbonate chemistry identical to that used for acclimation. For each treatment, pH_cyt_ was measured on a minimum of three independent days. To measure the response of cells to changes in external pH (pH_o_), acclimated cells were loaded with SNARF-1 in control ASW at pH 8.15. pH_o_ was then changed by consecutively perfusing the cells with ASW of pH 6.55, 7.55 and 8.75 for 5-min time intervals (flow-through ∼3 mL⋅min^−1^ in 0.5-mL total volume). In a final step, cells were washed with ASW of pH 8.15 to determine the pH_cyt_ drift between the beginning and the end of the experiment. If the pH_cyt_ offset was >4%, measurements were discarded from analysis.

For image processing, the mean fluorescence emission ratio (F_630_/F_580_) was determined using a region of interest encompassing the whole cell. We were unable to achieve a satisfactory in vivo calibration for SNARF-loaded cells using the nigericin technique, as we found that dye fluorescence was not stable after the addition of this protonophore. We therefore used an in vitro calibration, measuring the fluorescence emission ratio (F_630_/F_580_) of SNARF-1 (40 µM) in buffer (130 mM KC1, l mM MgCl_2_, 15 mM Hepes) of a range of pH (pH 6.75 to 7.5). From the calibration curve, the following relation was obtained (*R*^2^ = 0.86):

$${\text{pH}}_{\text{cyt}}=\left(0.8205\times \text{ln}\left({\text{F}}_{630}/{\text{F}}_{580}\right)\right)+7.1101.$$

### qPCR Analysis of Gene Expression.

qPCR was performed for *HV1* and *HV2* in cultures acclimated to pH 7.55, 8.15, and 8.75 (in triplicates). The expression of two endogenous reference genes (ERGs), *EFL* and *RPS1*, was measured alongside expression of the two target genes. Primers were designed to amplify products ∼150 bp in length. Primer quality was tested by performing efficiency curves for serial dilutions (1:5) of each primer pair (efficiencies were >98%, *R*^2^ values >0.96). RNA was extracted from *C. braarudii* cells using the Isolate II RNA Mini Kit (Bioline; Meridian Bioscience) with on-column DNA digestion. Thirty milliliters of exponential growth phase culture (∼4,000 cells⋅mL^−1^) was centrifuged at 3,800 × *g* for 5 min at 4 °C. Quality and quantity of extracted RNA were tested using a Nanodrop 1000 (Thermo Fisher Scientific) (A_260_/A_280_ ratios >1.80). Complementary DNA (cDNA) was synthesized from 50 ng RNA using a SensiFAST cDNA Synthesis Kit (Bioline), with a combination of random hexamers and oligo dT primers. No reverse-transcriptase controls (NRTCs) were generated to ensure no DNA contamination had occurred. qPCR runs were performed using a Rotorgene 6000 cycler (Qiagen). Each reaction (20 μL) consisted of 1 μL of cDNA substrate and 19 μL of a SensiFAST No-ROX Kit Master Mix (Bioline). Following primer optimization, 0.4 μM primer were used for all genes. PCR cycles were run with Rotorgene Q series software, comprising an initial 95 °C 2-min hold period, 40 cycles of 95 °C denaturing for 5 s, 62 °C annealing for 10 s, and 72 °C extension step (acquisition at end of extension step) for 20 s. A high resolution melt curve, 72 to 95 °C with 1 °C ramp, was conducted after amplification to ensure all amplicons had comparable melting temperatures.

For each sample, 1 μL of cDNA was analyzed in technical triplicates (target genes) or duplicates (reference genes). One qPCR plate contained all *HV1* or *HV2* primer reactions (or the NRTCs), as well as the ERG reactions (*EFL* and *RPS1*), control reactions (= *HV1* expressed in the pH 8.15 acclimation), no template controls, and two positive controls. Stability of the ERG was tested using geNorm (81). qPCR data were analyzed using an efficiency-corrected DDCt method, normalizing to the geometric mean of the two ERGs (81). Expression of *EFL* in all NRTC was at least 10 Ct smaller than the sample.

### Phylogenetic Analyses.

Previously identified Hv1 sequences from coccolithophores (21) were used as queries for sequence similarity searches of the available haptophyte transcriptomes within the Marine Microbial Eukaryote Sequencing Project (46, 82) (reassembled reads NCBI accession no. PRJEB37117). Further H_v_ sequences from other representative protist lineages were obtained from the Joint Genome Institute (https://phycocosm.jgi.doe.gov/phycocosm/home). Protist H_v_ sequences possess an extended extracellular loop between transmembrane domains S1 and S2 (21, 45), enabling the generation of a longer multiple sequence alignment and improved resolution of the haptophyte H_v_ sequences. H_v_ sequences from other lineages (e.g., animals) lack the extended S1–S2 loop, although phylogenetic trees constructed with a wider range of eukaryotes exhibited a similar topology. Potential H_v_ sequences identified by sequence similarity searches were manually inspected using a multiple sequence alignment to assess the presence of conserved residues essential for H^+^ channel function (23). The multiple sequence alignments were then refined using GBLOCKS 0.91b to remove poorly aligned residues (83). Phylogenetic analysis was performed using the maximum likelihood method within MEGAX software (84) after prior determination of the best substitution model (WAG with gamma and invariant).

### Statistical Analyses.

For coccolith morphologies, the mean and SE values were calculated from experimental replicates with a minimum of 350 coccoliths scored for each replicate. For electrophysiology, the means and SE values were calculated from individual replicate cells from each treatment, with *n* numbers given in each figure. For intracellular pH measurements, differences in pH between treatments were tested with one-way ANOVA Holm–Sidak post hoc tests. Differences in distribution of pH values between treatments were assessed with two-sample Kolmogorov–Smirnov tests.

## Supplementary Material

Supplementary File

## Data Availability

Image data PDF, Excel, and Sigmaplot files of electrophysiological data, Excel files of cellular pH data, Excel files of growth and cell composition data, and Excel files of gene expression data have been deposited in the DASSH data archive (DOI: 10.17031/1824) (85).

## References

[1] S. C. Doney, V. J. Fabry, R. A. Feely, J. A. Kleypas, Ocean acidification: The other CO_2_ problem. Annu. Rev. Mar. Sci. 1, 169–192 (2009). 10.1146/annurev.marine.010908.163834 21141034

[2] B. Rost, I. Zondervan, D. Wolf-Gladrow, Sensitivity of phytoplankton to future changes in ocean carbonate chemistry: Current knowledge, contradictions and research directions. Mar. Ecol. Prog. Ser. 373, 227–237 (2008).

[3] L. Q. Jiang, B. R. Carter, R. A. Feely, S. K. Lauvset, A. Olsen, Surface ocean pH and buffer capacity: Past, present and future. Sci. Rep. 9, 18624 (2019). 31819102 10.1038/s41598-019-55039-4 PMC6901524

[4] G. E. Hofmann , High-frequency dynamics of ocean pH: A multi-ecosystem comparison. PLoS One 6, e28983 (2011). 22205986 10.1371/journal.pone.0028983 PMC3242773

[5] J. T. Wootton, C. A. Pfister, J. D. Forester, Dynamic patterns and ecological impacts of declining ocean pH in a high-resolution multi-year dataset. Proc. Natl. Acad. Sci. U.S.A. 105, 18848–18853 (2008). 19033205 10.1073/pnas.0810079105 PMC2596240

[6] D. Capone, D. Hutchins, Microbial biogeochemistry of coastal upwelling regimes in a changing ocean. Nat. Geosci. 6, 711–717 (2013).

[7] G. G. Waldbusser, J. E. Salisbury, Ocean acidification in the coastal zone from an organism’s perspective: Multiple system parameters, frequency domains, and habitats. Annu. Rev. Mar. Sci. 6, 221–247 (2014). 10.1146/annurev-marine-121211-172238 23987912

[8] W. M. Balch, The ecology, biogeochemistry, and optical properties of Coccolithophores. Annu. Rev. Mar. Sci. 10, 71–98 (2018). 10.1146/annurev-marine-121916-063319 29298138

[9] W. M. Balch , Coccolithophore distributions of the North and South Atlantic Ocean. Deep Sea Res. Part I Oceanogr. Res. Pap. 151, 103066 (2019).

[10] L. T. Bach, U. Riebesell, M. A. Gutowska, L. Federwisch, K. G. Schulz, A unifying concept of coccolithophore sensitivity to changing carbonate chemistry embedded in an ecological framework. Prog. Oceanogr. 135, 125–138 (2015).

[11] N. A. Gafar, B. D. Eyre, K. G. Schulz, Particulate inorganic to organic carbon production as a predictor for coccolithophorid sensitivity to ongoing ocean acidification. Limnol. Oceanogr. Lett. 4, 62–70 (2019).

[12] K. J. Kroeker , Impacts of ocean acidification on marine organisms: Quantifying sensitivities and interaction with warming. Glob. Change Biol. 19, 1884–1896 (2013). 10.1111/gcb.12179 PMC3664023 23505245

[13] J. Meyer, U. Riebesell, Reviews and syntheses: Responses of coccolithophores to ocean acidification: A meta-analysis. Biogeosciences 12, 1671–1682 (2015).

[14] J. A. Raven, K. Crawfurd, Environmental controls on coccolithophore calcification. Mar. Ecol. Prog. Ser. 470, 137–166 (2012).

[15] G. Langer , Species-specific responses of calcifying algae to changing seawater carbonate chemistry. Geochem. Geophys. Geosyst. 7, 10.1029/2005GC001227 (2006).

[16] L. T. Bach , Dissecting the impact of CO_2_ and pH on the mechanisms of photosynthesis and calcification in the coccolithophore Emiliania huxleyi. New Phytol. 199, 121–134 (2013). 23496417 10.1111/nph.12225

[17] C. S. Sikes, R. D. Roer, K. M. Wilbur, Photosynthesis and coccolith formation - inorganic carbon-sources and net inorganic reaction of deposition. Limnol. Oceanogr. 25, 248–261 (1980).

[18] A. R. Taylor, C. Brownlee, G. Wheeler, Coccolithophore cell biology: Chalking up progress. Annu. Rev. Mar. Sci. 9, 283–310 (2017). 10.1146/annurev-marine-122414-034032 27814031

[19] L. T. Bach, C. Bauke, K. J. S. Meier, U. Riebesell, K. G. Schulz, Influence of changing carbonate chemistry on morphology and weight of coccoliths formed by Emiliania huxleyi. Biogeosciences 9, 3449–3463 (2012).

[20] L. T. Bach, U. Riebesell, K. G. Schulz, Distinguishing between the effects of ocean acidification and ocean carbonation in the coccolithophore Emiliania huxleyi. Limnol. Oceanogr. 56, 2040–2050 (2011).

[21] A. R. Taylor, A. Chrachri, G. Wheeler, H. Goddard, C. Brownlee, A voltage-gated H^+^ channel underlying pH homeostasis in calcifying coccolithophores. PLoS Biol. 9, e1001085 (2011). 21713028 10.1371/journal.pbio.1001085 PMC3119654

[22] K. Suffrian, K. G. Schulz, M. A. Gutowska, U. Riebesell, M. Bleich, Cellular pH measurements in Emiliania huxleyi reveal pronounced membrane proton permeability. New Phytol. 190, 595–608 (2011). 21294736 10.1111/j.1469-8137.2010.03633.x

[23] T. E. DeCoursey, Voltage-gated proton channels: Molecular biology, physiology, and pathophysiology of the H(V) family. Physiol. Rev. 93, 599–652 (2013). 23589829 10.1152/physrev.00011.2012 PMC3677779

[24] J. Fresnel, I. Probert, The ultrastructure and life cycle of the coastal coccolithophorid Ochrosphaera neapolitana (Prymnesiophyceae). Eur. J. Phycol. 40, 105–122 (2005).

[25] Y. W. Liu, R. A. Eagle, S. M. Aciego, R. E. Gilmore, J. B. Ries, A coastal coccolithophore maintains pH homeostasis and switches carbon sources in response to ocean acidification. Nat. Commun. 9, 2857 (2018). 30030435 10.1038/s41467-018-04463-7 PMC6054640

[26] A. G. Saez, A. Zaldivar-Riveron, L. K. Medlin, Molecular systematics of the Pleurochrysidaceae, a family of coastal coccolithophores (Haptophyta). J. Plankton Res. 30, 559–566 (2008).

[27] M. M. White , Calcification of an estuarine coccolithophore increases with ocean acidification when subjected to diurnally fluctuating carbonate chemistry. Mar. Ecol. Prog. Ser. 601, 59–76 (2018).

[28] C. J. Daniels , Species-specific calcite production reveals Coccolithus pelagicus as the key calcifier in the Arctic Ocean. Mar. Ecol. Prog. Ser. 555, 29–47 (2016).

[29] C. J. Daniels, R. M. Sheward, A. J. Poulton, Biogeochemical implications of comparative growth rates of Emiliania huxleyi and Coccolithus species. Biogeosciences 11, 6915–6925 (2014).

[30] A. S. R. Hernandez , Coccolithophore biodiversity controls carbonate export in the Southern Ocean. Biogeosciences 17, 245–263 (2020).

[31] S. A. Krug, K. G. Schulz, U. Riebesell, Effects of changes in carbonate chemistry speciation on Coccolithus braarudii: A discussion of coccolithophorid sensitivities. Biogeosciences 8, 771–777 (2011).

[32] M. N. Muller, K. G. Schulz, U. Riebesell, Effects of long-term high CO_2_ exposure on two species of coccolithophores. Biogeosciences 7, 1109–1116 (2010).

[33] D. M. Kottmeier, S. D. Rokitta, B. Rost, H^+^-driven increase in CO_2_ uptake and decrease in HCO_3_^-^ uptake explain coccolithophores’ acclimation responses to ocean acidification. Limnol. Oceanogr. 61, 2045–2057 (2016).

[34] D. M. Kottmeier, S. D. Rokitta, P. D. Tortell, B. Rost, Strong shift from HCO_3_ (-) to CO_2_ uptake in Emiliania huxleyi with acidification: New approach unravels acclimation versus short-term pH effects. Photosynth. Res. 121, 265–275 (2014). 24563097 10.1007/s11120-014-9984-9 PMC4077253

[35] M. Cachao, M. T. Moita, Coccolithus pelagicus, a productivity proxy related to moderate fronts off Western Iberia. Mar. Micropaleontol. 39, 131–155 (2000).

[36] J. Giraudeau, P. M. S. Monteiro, K. Nikodemus, Distribution and malformation of living coccolithophores in the northern Benguela upwelling system off Namibia. Mar. Micropaleontol. 22, 93–110 (1993).

[37] G. Faucher, U. Riebesell, L. T. Bach, Can morphological features of coccolithophores serve as a reliable proxy to reconstruct environmental conditions of the past? Clim. Past 16, 1007–1025 (2020).

[38] U. Riebesell , Reduced calcification of marine plankton in response to increased atmospheric CO_2_. Nature 407, 364–367 (2000). 11014189 10.1038/35030078

[39] G. M. Durak , A role for diatom-like silicon transporters in calcifying coccolithophores. Nat. Commun. 7, 10543 (2016). 26842659 10.1038/ncomms10543 PMC4742977

[40] A. C. Gerecht, L. Supraha, B. Edvardsen, G. Langer, J. Henderiks, Phosphorus availability modifies carbon production in Coccolithus pelagicus (Haptophyta). J. Exp. Mar. Biol. Ecol. 472, 24–31 (2015).

[41] G. Langer, K. Oetjen, T. Brenneis, On culture artefacts in coccolith morphology. Helgol. Mar. Res. 67, 359–369 (2013).

[42] A. R. Taylor, C. Brownlee, A novel Cl^-^ inward-rectifying current in the plasma membrane of the calcifying marine phytoplankton Coccolithus pelagicus. Plant Physiol. 131, 1391–1400 (2003). 12644688 10.1104/pp.011791 PMC166898

[43] A. R. Taylor, C. Brownlee, G. L. Wheeler, Proton channels in algae: Reasons to be excited. Trends Plant Sci. 17, 675–684 (2012). 22819465 10.1016/j.tplants.2012.06.009

[44] G. Kigundu, J. L. Cooper, S. M. E. Smith, Hv1 proton channels in dinoflagellates: Not just for bioluminescence? J. Eukaryot. Microbiol. 65, 928–933 (2018). 29698585 10.1111/jeu.12627 PMC7167071

[45] J. D. Rodriguez , Identification of a vacuolar proton channel that triggers the bioluminescent flash in dinoflagellates. PLoS One 12, e0171594 (2017). 28178296 10.1371/journal.pone.0171594 PMC5298346

[46] P. J. Keeling , The Marine Microbial Eukaryote Transcriptome Sequencing Project (MMETSP): Illuminating the functional diversity of eukaryotic life in the oceans through transcriptome sequencing. PLoS Biol. 12, e1001889 (2014). 24959919 10.1371/journal.pbio.1001889 PMC4068987

[47] N. A. Nimer, C. Brownlee, M. J. Merrett, Carbon-dioxide availability, intracellular pH and growth-rate of the coccolithophore Emiliania huxleyi. Mar. Ecol. Prog. Ser. 109, 257–262 (1994).

[48] T. E. Decoursey, Voltage-gated proton channels and other proton transfer pathways. Physiol. Rev. 83, 475–579 (2003). 12663866 10.1152/physrev.00028.2002

[49] L. Hong, I. H. Kim, F. Tombola, Molecular determinants of Hv1 proton channel inhibition by guanidine derivatives. Proc. Natl. Acad. Sci. U.S.A. 111, 9971–9976 (2014). 24912149 10.1073/pnas.1324012111 PMC4103315

[50] L. Hong, M. M. Pathak, I. H. Kim, D. Ta, F. Tombola, Voltage-sensing domain of voltage-gated proton channel Hv1 shares mechanism of block with pore domains. Neuron 77, 274–287 (2013). 23352164 10.1016/j.neuron.2012.11.013 PMC3559007

[51] V. T. Lim, J. A. Freites, F. Tombola, D. J. Tobias, Thermodynamics and mechanism of the membrane permeation of Hv1 channel blockers. J. Membr. Biol. 254, 5–16 (2021). 33196887 10.1007/s00232-020-00149-8 PMC8549822

[52] J. Kalia, K. J. Swartz, Elucidating the molecular basis of action of a classic drug: Guanidine compounds as inhibitors of voltage-gated potassium channels. Mol. Pharmacol. 80, 1085–1095 (2011). 21926190 10.1124/mol.111.074989 PMC3228538

[53] K. E. Helliwell , A novel single-domain Na^+^-selective voltage-gated channel in photosynthetic eukaryotes. Plant Physiol. 184, 1674–1683 (2020). 33004614 10.1104/pp.20.00889 PMC7723092

[54] L. Schlüter, K. T. Lohbeck, J. P. Gröger, U. Riebesell, T. B. Reusch, Long-term dynamics of adaptive evolution in a globally important phytoplankton species to ocean acidification. Sci. Adv. 2, e1501660 (2016). 27419227 10.1126/sciadv.1501660 PMC4942326

[55] S. Tong, K. Gao, D. A. Hutchins, Adaptive evolution in the coccolithophore Gephyrocapsa oceanica following 1,000 generations of selection under elevated CO_2_. Glob. Change Biol. 24, 3055–3064 (2018). 10.1111/gcb.14065 29356310

[56] X. A. Padin, A. Velo, F. F. Pérez, ARIOS: A database for ocean acidification assessment in the Iberian upwelling system (1976–2018). Earth Syst. Sci. Data 12, 2647–2663 (2020).

[57] N. Gruber, P. W. Boyd, T. L. Frölicher, M. Vogt, Biogeochemical extremes and compound events in the ocean. Nature 600, 395–407 (2021). 34912083 10.1038/s41586-021-03981-7

[58] S. Fiorini, J. J. Middelburg, J. P. Gattuso, Effects of elevated CO_2_ partial pressure and temperature on the coccolithophore Syracosphaera pulchra. Aquat. Microb. Ecol. 64, 221–232 (2011).

[59] M. Hermoso, F. Minoletti, Mass and fine-scale morphological changes induced by changing seawater pH in the coccolith Gephyrocapsa oceanica. J. Geophys. Res. Biogeosci. 123, 2761–2774 (2018).

[60] A. S. Rigual-Hernández , Full annual monitoring of Subantarctic Emiliania huxleyi populations reveals highly calcified morphotypes in high-CO_2_ winter conditions. Sci. Rep. 10, 2594 (2020). 32054880 10.1038/s41598-020-59375-8 PMC7018777

[61] H. L. McClelland , Calcification response of a key phytoplankton family to millennial-scale environmental change. Sci. Rep. 6, 34263 (2016). 27677230 10.1038/srep34263 PMC5039703

[62] P. von Dassow , Over-calcified forms of the coccolithophore Emiliania huxleyi in high-CO_2_ waters are not preadapted to ocean acidification. Biogeosciences 15, 1515–1534 (2018).

[63] B. Hönisch , The geological record of ocean acidification. Science 335, 1058–1063 (2012). 22383840 10.1126/science.1208277

[64] F. M. Monteiro , Why marine phytoplankton calcify. Sci. Adv. 2, e1501822 (2016). 27453937 10.1126/sciadv.1501822 PMC4956192

[65] M. J. Henehan , Rapid ocean acidification and protracted Earth system recovery followed the end-Cretaceous Chicxulub impact. Proc. Natl. Acad. Sci. U.S.A. 116, 22500–22504 (2019). 31636204 10.1073/pnas.1905989116 PMC6842625

[66] P.R. Bown, J.A. Lees, J.R. Young, “Calcareous nannoplankton evolution and diversity through time” in Coccolithophores: From Molecular Processes to Global Impact, H. R. Thierstein, J. R. Young, Eds. (Springer, 2004), pp. 481–508.

[67] S. J. Gibbs , Algal plankton turn to hunting to survive and recover from end-Cretaceous impact darkness. Sci. Adv. 6, eabc9123 (2020). 33127682 10.1126/sciadv.abc9123 PMC7608818

[68] K. Hagino , Re-discovery of a “living fossil” coccolithophore from the coastal waters of Japan and Croatia. Mar. Micropaleontol. 116, 28–37 (2015).

[69] D. B. Van de Waal, E. Litchman, Multiple global change stressor effects on phytoplankton nutrient acquisition in a future ocean. Philos. Trans. R. Soc. Lond. B. Biol. Sci. 375, 20190706 (2020). 32200734 10.1098/rstb.2019.0706 PMC7133525

[70] M. J. Frada, E. M. Bendif, S. Keuter, I. Probert, The private life of coccolithophores. Perspect. Phycol. 6, 11–30 (2018).

[71] L. Berry, A. R. Taylor, U. Lucken, K. P. Ryan, C. Brownlee, Calcification and inorganic carbon acquisition in coccolithophores. Funct. Plant Biol. 29, 289–299 (2002). 32689476 10.1071/PP01218

[72] Y. Y. Xu, D. Pierrot, W. J. Cai, Ocean carbonate system computation for anoxic waters using an updated CO2SYS program. Mar. Chem. 195, 90–93 (2017).

[73] D. M. Kottmeier, A. Terbruggen, D. A. Wolf-Gladrow, S. Thoms, Diel variations in cell division and biomass production of Emiliania huxleyi—Consequences for the calculation of physiological cell parameters. Limnol. Oceanogr. 65, 1781–1800 (2020).

[74] S. Menden-Deuer, E. J. Lessard, Carbon to volume relationships for dinoflagellates, diatoms, and other protist plankton. Limnol. Oceanogr. 45, 569–579 (2000).

[75] J. R. Young, P. Ziveri, Calculation of coccolith volume and its use in calibration of carbonate flux estimates. Deep Sea Res. Part II Top. Stud. Oceanogr. 47, 9–11 (2000).

[76] G. Langer , Role of silicon in the development of complex crystal shapes in coccolithophores. New Phytol. 231, 1845–1857 (2021). 33483994 10.1111/nph.17230

[77] J. R. Young, P. Westbroek, Genotypic variation in the coccolithophorid species Emiliania huxleyi. Mar. Micropaleontol. 18, 5–23 (1991).

[78] G. Langer, I. Benner, Effect of elevated nitrate concentration on calcification in Emiliania huxleyi. J. Nannoplankton Res. 30, 77–80 (2009).

[79] V. V. Cherny, T. E. DeCoursey, pH-dependent inhibition of voltage-gated H(+) currents in rat alveolar epithelial cells by Zn(2+) and other divalent cations. J. Gen. Physiol. 114, 819–838 (1999). 10578017 10.1085/jgp.114.6.819 PMC2230650

[80] A. Krężel, W. Maret, The biological inorganic chemistry of zinc ions. Arch. Biochem. Biophys. 611, 3–19 (2016). 27117234 10.1016/j.abb.2016.04.010 PMC5120989

[81] J. Vandesompele , Accurate normalization of real-time quantitative RT-PCR data by geometric averaging of multiple internal control genes. Genome Biol. 3, research0034 (2002). 12184808 10.1186/gb-2002-3-7-research0034 PMC126239

[82] L. K. Johnson, H. Alexander, C. T. Brown, Re-assembly, quality evaluation, and annotation of 678 microbial eukaryotic reference transcriptomes. Gigascience 8, giy158 (2019). 30544207 10.1093/gigascience/giy158 PMC6481552

[83] G. Talavera, J. Castresana, Improvement of phylogenies after removing divergent and ambiguously aligned blocks from protein sequence alignments. Syst. Biol. 56, 564–577 (2007). 17654362 10.1080/10635150701472164

[84] S. Kumar, G. Stecher, M. Li, C. Knyaz, K. Tamura, MEGA X: Molecular evolutionary genetics analysis across computing platforms. Mol. Biol. Evol. 35, 1547–1549 (2018). 29722887 10.1093/molbev/msy096 PMC5967553

[85] C. Brownlee, Coccolithophore experimental data for Kottmeier et al Proc. Natl. Acad. Sci. 2022. The Archive for Marine Species and Habitats Data (DASSH). https://www.dassh.ac.uk/doitool/data/1824. Deposited 5 April 2022.

