# Supplementary material for: Reduced H+ channel activity disrupts pH homeostasis and calcification in coccolithophores at low ocean pH
Source: Proc Natl Acad Sci U S A. 2022 May 6;119(19):e2118009119. doi: 10.1073/pnas.2118009119 (PMC9171652; doi:10.1073/pnas.2118009119)
Supplement: Supplementary File [file pnas.2118009119.sapp.pdf]

Reduced H^+ channel activity disrupts pH homeostasis and calcification in coccolithophores at low ocean pH

Dorothee Kottmeier[1,3], Abdesslam Chrachri[1], Gerald Langer[1], Katherine Helliwell[1,4], Glen L. Wheeler[1*], Colin Brownlee[1,2*]

SI Appendix

Figs. S 1-10

Tables S 1-4

Text S 1

References

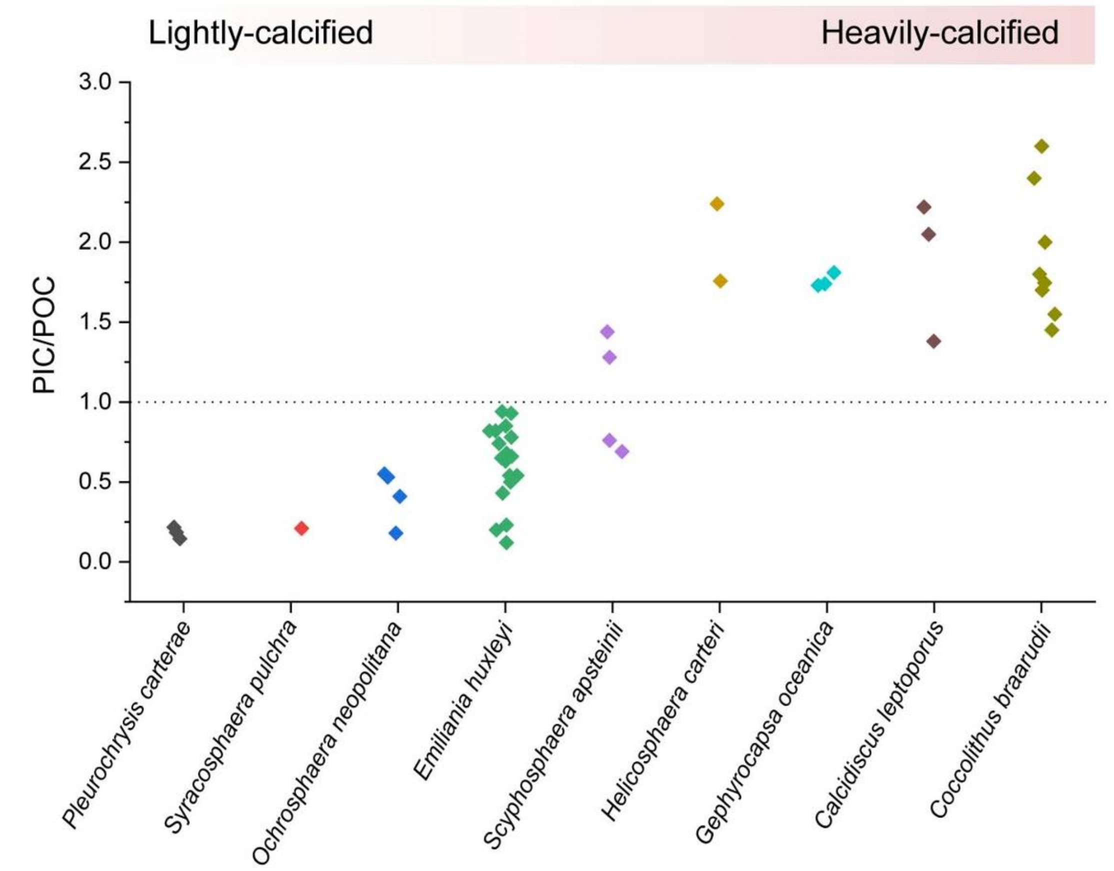

Fig. S1: Coccolithophore species exhibit different degrees of calcification. The graph shows the ratio of particulate inorganic carbon to particulate organic carbon measured from a range of different coccolithophore species. The data were collated from a range of studies, but do not represent an exhaustive list of all studies. In studies where carbonate chemistry was manipulated, only the PIC/POC values for cultures similar to present day carbonate chemistry scenarios have been used (pH 8.05-8.30). The data indicate that the H^+ load associated with intracellular calcification is likely to differ markedly between species. Note that freshly isolated *E. huxleyi* strains typically exhibit a PIC/POC close to 1 (1). The much lower PIC/POC values in some *E. huxleyi* strains in our analysis above likely reflects *E. huxleyi* isolates that exhibit a reduced ability to calcify following prolonged maintenance in laboratory culture. Data were taken from (2-14).

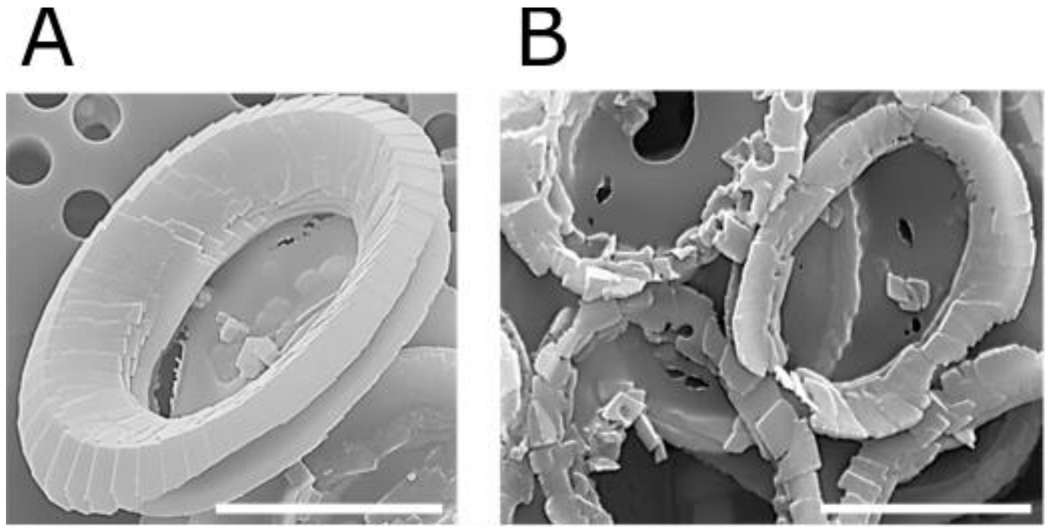

Fig. S2. Morphological distinction between incomplete and type-pH malformations

A) Scanning electron micrograph displaying incomplete coccolith. Note that the individual elements are shorter, but not malformed. Bar = 5 µm. B) Type-pH malformation showing the individual elements are not only shorter than a mature coccolith, but display extensive malformations. Bar = 5 µm.

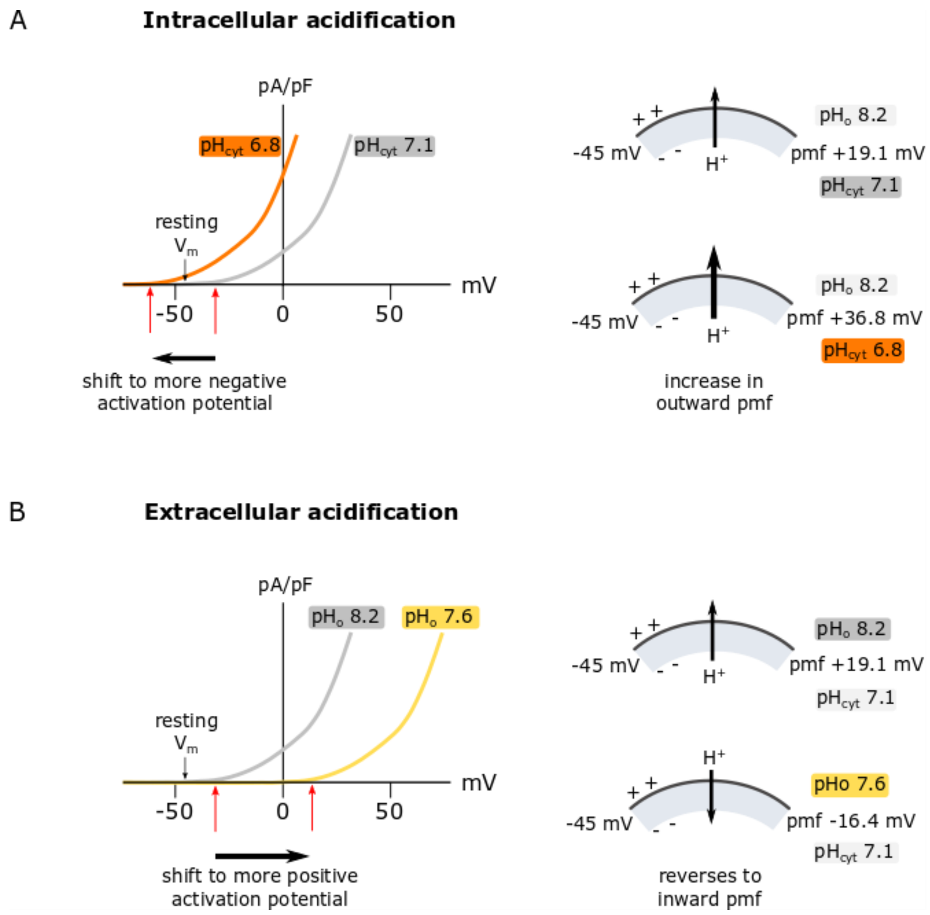

Fig. S3: The impact of changes in the transmembrane H^+ gradient on the operation of voltage-gated H^+ channels. A) A small decrease in cytosolic pH (pH_{cyt}) due to the intracellular production of H^+ has a twofold effect on H^+ channel operation. It shifts the activation potential more negative, increasing the open probability at resting membrane potential (V_m) (15), and also increases the transmembrane H^+ gradient, which results in a larger outward proton motive force (pmf). These two effects combine to support H^+ efflux and restore pH_{cyt}. B) If the extracellular pH is lower, as in an ocean acidification scenario, the opposite occurs. The activation potential of the H^+ channel is shifted to a more positive membrane potential and the pmf is reversed to a net inward pmf. In this scenario, small decreases in pH_{cyt} are unlikely to activate the H^+ channel and the pmf is unfavourable for H^+ efflux. Thus, H^+ efflux through the H^+ channel is unlikely to be an effective mechanism

of pH regulation. Note also that the pH-dependent shift in activation voltage means that inward H^+ flux through the H^+ channel is unlikely.

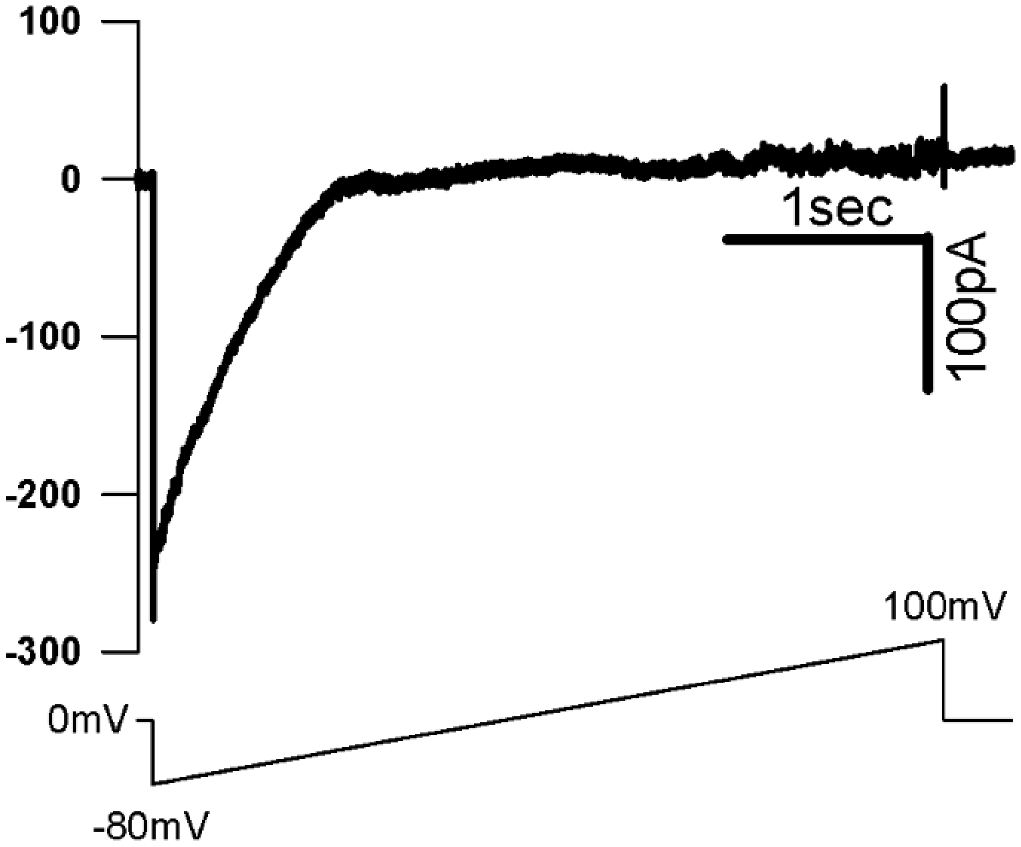

Fig. S4: *C. braarudii* cells without an outward H^+ current still exhibit functional Cl^- currents.

Representative trace of a *C. braarudii* cell acclimated to pH 7.55. *Top trace:* The presence of the large inward rectifying Cl^- current is clearly visible at negative membrane potentials but no outward current is apparent at positive potentials. *Bottom trace:* Using the patch-clamp technique, a voltage ramp (from -80 to + 100 mV from a holding potential of 0 mV) was applied to the membrane to monitor both inward and outward current.

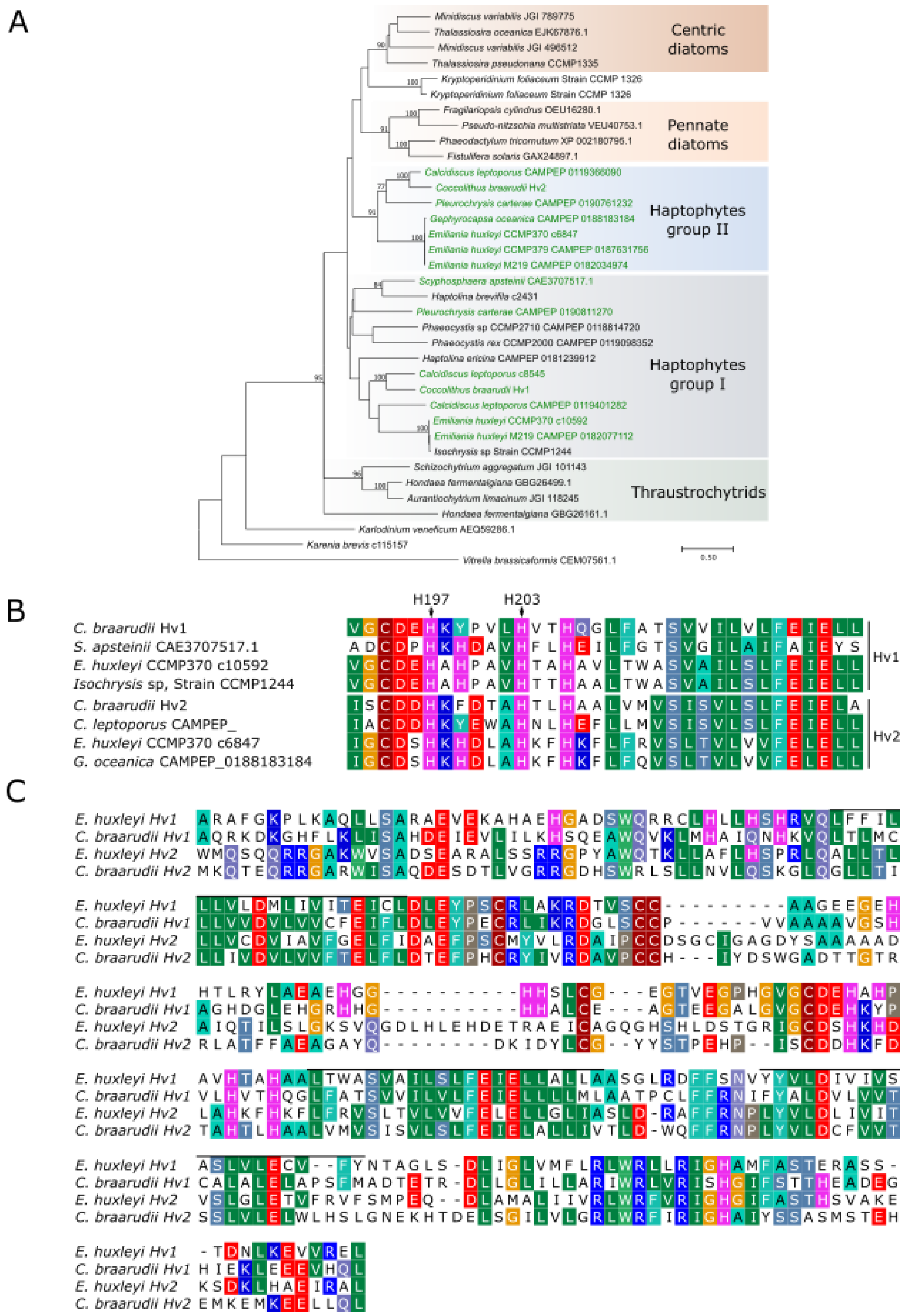

Fig. S5: Coccolithophores possess multiple H_v homologues. **(A)** A phylogenetic tree of haptophyte H_v homologues. A multiple sequence alignment of haptophyte H_v sequences was constructed using eukaryote H_v sequences. Protist sequences have an extended extracellular loop between transmembrane domains S1 and S2, allowing for a longer multiple sequence alignment. Green text indicates calcified haptophyte lineages.

Coccolithophore H_v sequences fall into two major groups. Group I contains H_v sequences from all haptophytes (including non-calcified haptophytes), including previously characterised Hv1 sequences from *E. huxleyi* and *C. braarudii* (ADM25825.1) (15). Group II represents a well-supported clade containing only sequences from calcified coccolithophore species. This distribution suggests that Group I sequences (Hv1) could perform a general role in haptophyte physiology (e.g. in supporting NADPH oxidase activity (16), whereas the restriction of Group II (Hv2) sequences to calcified coccolithophores suggests that they could play specialised roles in the calcification process. The tree was constructed using the maximum likelihood method with the WAG substitution model with gamma and invariant. Numbers above nodes indicate bootstrap support (100 bootstraps were performed, values>70 are shown). The final alignment size was 149 amino acids. **(B)** Multiple sequence alignment of coccolithophore H_v sequences showing that the histidine residues (arrowed) responsible for Zn inhibition of *E. huxleyi* Hv1 (15) are strongly conserved. **(C)** Multiple sequence alignment of *E. huxleyi* and *C. braarudii* H_v sequences showing approximate position of the four transmembrane domains (black lines). Accession numbers are as follows: *E. huxleyi* M219 Hv1 CAMPEP_0182077112, Hv2 CAMPEP_0182034974; *C. braarudii* Hv1 ADM25825.1, Hv2 CAD8615310.1.

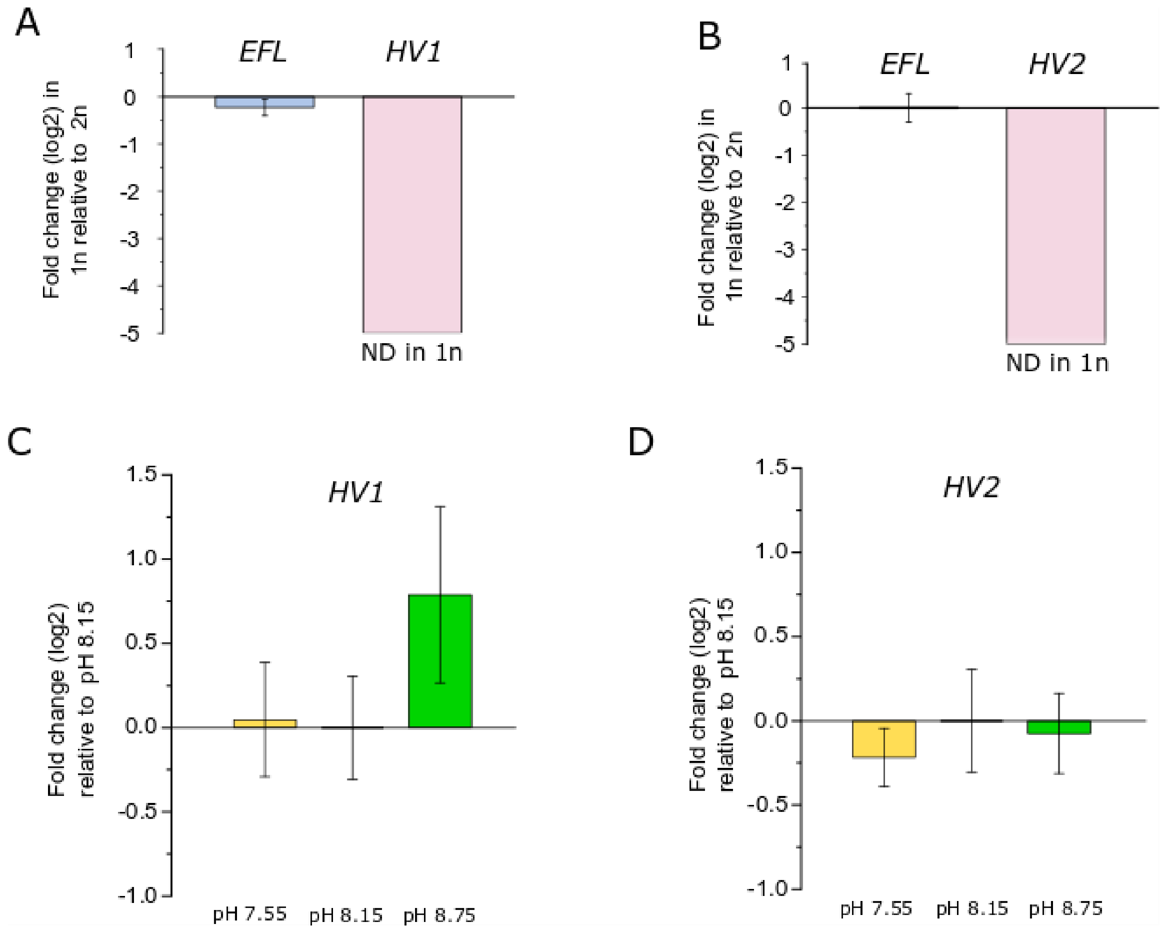

Fig. S6: Gene expression of *C. braarudii* H^+ channels determined by qPCR. A) Expression of *HV1* in the haploid (1n) and diploid (2n) life cycle phases of *C. braarudii*. The haploid is very lightly calcified while the diploid is heavily calcified. Expression of Hv1 was normalised to a single reference gene (*RPS*) and is shown as the fold change in 1n relative to 2n. *HV1* was not detected (ND) in 1n cells. The expression of the *EFL* reference gene is also shown (normalised to *RPS*) to show that the expression of reference genes did not differ markedly between life cycle phases. **B)** Expression of *HV2* in the haploid (1n) and diploid (2n) life cycle phases. Expression of *HV2* was normalised to a single reference gene (*RPS*) and is shown as the fold change in 1n relative to 2n. *HV2* was not detected in 1n cells. In all cases n=3 biological replicates, error bars represent SE. **C)** Expression of *HV1* in *C. braarudii* cells acclimated to pH 7.55, 8.15 and 8.75. Expression

was normalised to two reference genes (*EFL* and *RPS*) and is shown as fold change relative to the expression levels of *HV1* in pH 8.15 acclimated cells. No significant differences were found (1-way ANOVA). **D)** Expression of *HV2*. Expression was normalised to two reference genes (*EFL* and *RPS*) and is shown as fold change relative to the expression levels of Hv1 in pH 8.15 acclimated cells. No significant differences were found (1-way ANOVA). In all cases error bars represent SE.

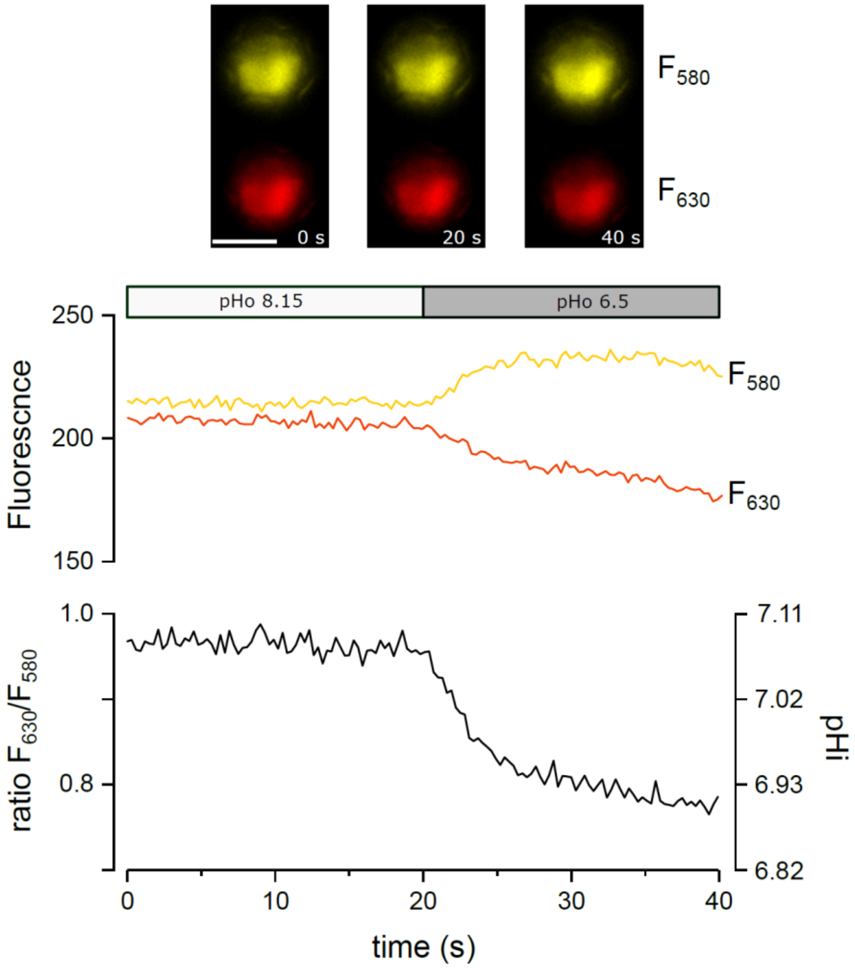

Fig. S7: Measurement of pH_{cyt} in *C. braarudii* using the fluorescent dye SNARF-1. *C. braarudii* cells were ester-loaded with the pH-responsive fluorescent dye SNARF-1 and viewed by epifluorescent microscopy. Cells showed an even distribution of dye in the cytoplasm, indicating that the dye had not entered other cellular compartments (which could have a different pH). Fluorescence emission was monitored at 580 and 630 nm. On switching the external pH from 8.15 to 6.55 by perfusion, F_{580} increases while F_{630} decreases, leading to an overall decrease in the F_{630}/F_{580} ratio. An *in vitro* calibration curve of SNARF-1 was used to estimate pH_{cyt} (see Methods). In the example shown, the cell exhibits a rapid decrease in pH_{cyt}, typical of *C. braarudii* cells following exposure to lower external pH. Bar = 10 µm

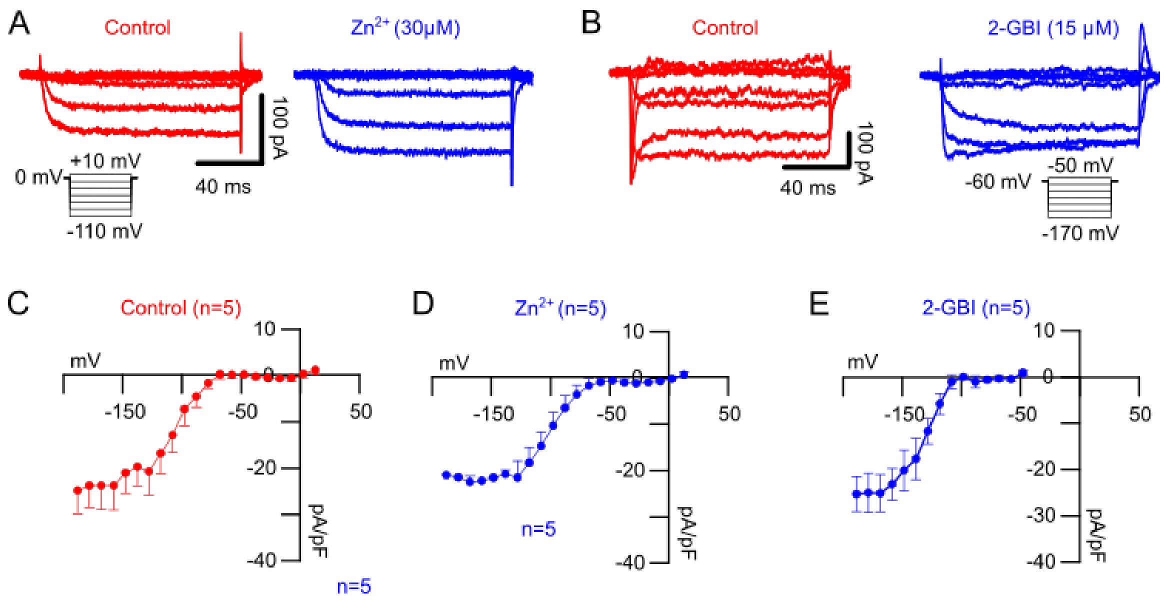

Fig S8: Cl⁻ currents in *C. braarudii* cells treated with H^+ channel inhibitors. A) Hyperpolarisation activates an inwardly-rectifying Cl^- current in *C. braarudii* that acts to regulate membrane potential. This current is inhibited by high concentrations of Zn^{2+} (>200 µM) (17), but there is little effect of Zn^{2+} at 30 µM. Typical currents activated by incremental 10 mV hyperpolarizing voltage pulses (20 mV increments are shown for clarity) in an untreated control cell (left) and a cell treated with 30 µM Zn^{2+} (right). **B)** Typical currents activated by a series of hyperpolarizing voltage pulses in an untreated control cell (left) and a cell treated with 15 µM 2-GBI for 4 h (right). **C)** Current-voltage curve for untreated control cells (n=5), error bars denote SE. **D)** Current-voltage curve for Zn^{2+}-treated cells (30 µM). **E)** Current-voltage curve for cells treated with 2-GBI (15 µM, 4 h).

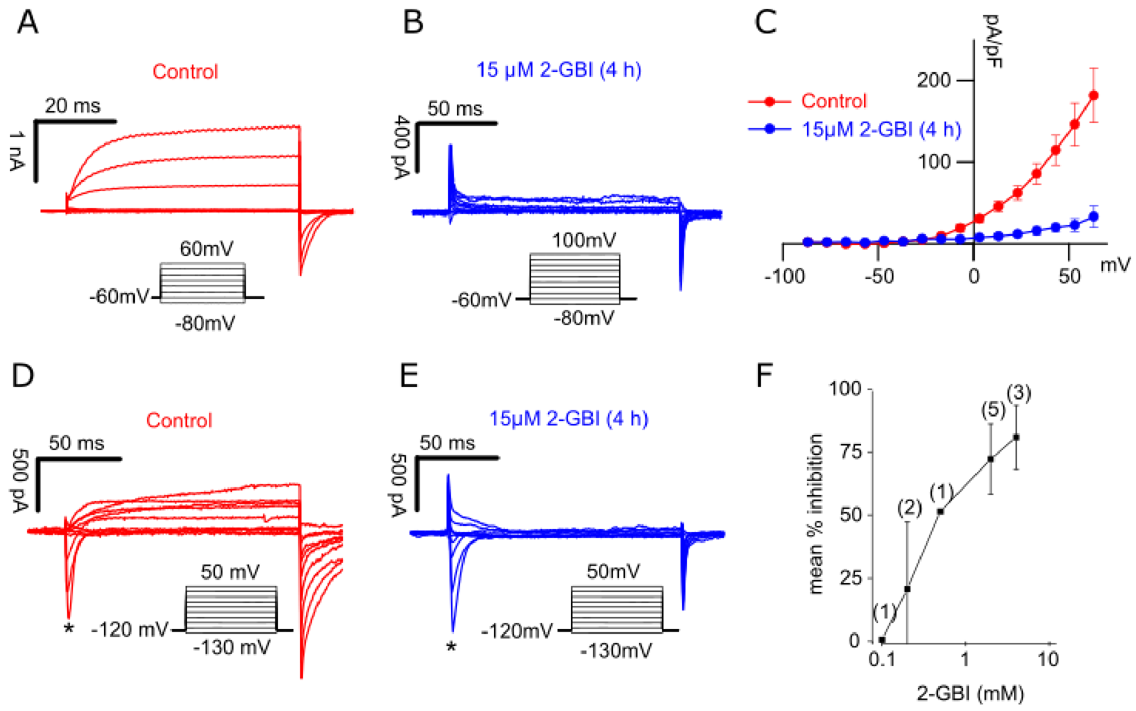

Fig. S9: Inhibition of H^+ currents in *C. braarudii* following prolonged exposure to 2-GBI. A) Whole cell currents from an untreated control *C. braarudii* cell in response to incremental 10 mV depolarisations from −80 to +60 mV (only 20 mV increments are shown for clarity). **B)** Inhibition of the outward H^+ currents following extracellular addition of the H_V inhibitor 2-guanidinobenzimidazole (2-GBI) at 15 μM for 4 h. **C)** Mean current voltage curves (± SE) for control (n=11) and 2-GBI-treated cells (15 μM for 4 h) (n=7). **D)** Whole cell currents from an untreated control cell held at a modified voltage protocol (holding potential -120 mV, incremental 10 mV depolarisations from −130 to +60 mV) to visualise the fast action potential of coccolithophores (17, 18). The fast activating and inactivating voltage-activated inward current is marked with an asterisk. **E)** The fast voltage-activated inward current (asterisk) is not inhibited in cells treated with 15 μM 2-GBI for 4 h. A representative trace is shown (n=7 cells examined). **F)** Dose response curve showing the effect of a much shorter exposure (< 5 minutes) to 2-GBI on outward H^+ currents in *C. braarudii* cells. 2-GBI acts by binding to the intracellular side of the voltage-sensing domain and therefore only has a limited effect on H^+ currents when applied

extracellularly at micromolar concentrations in short–term experiments (19). Outward currents in the presence of 0.1-4 mM 2-GBI were normalised to the untreated control currents to show percentage inhibition. Values in parentheses represent n. Error bars represent SE.

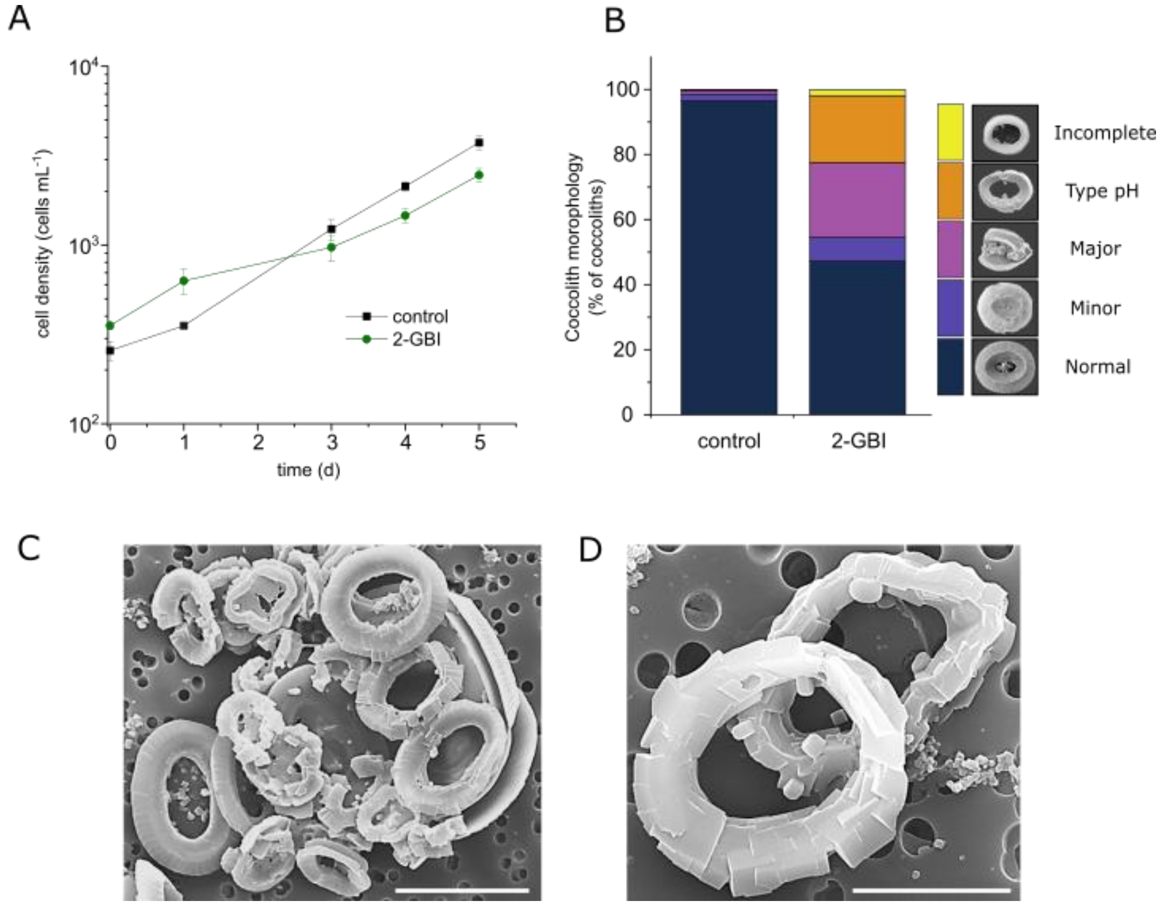

Fig S10: Effects of the H_v inhibitor 2-GBI on coccolith morphology in *C. braarudii*. **A)** Growth of *C. braarudii* cells in 2-GBI. Changes in cell density are shown in the presence of 15 µM 2-GBI at seawater pH of 8.15. n=3, error bars = SE. **B)** Quantitative analysis of coccolith morphology following treatment with 2-GBI. Coccoliths were categorised into morphological categories (see Materials and Methods). The counts represent the mean of three independent replicate treatments, with a minimum of 350 coccoliths were counted for each replicate. Cells exposed to 15 µM 2-GBI for 5 d exhibit a substantial increase in the proportion of the distinctive type-pH coccolith malformations, only observed at low pH or after treatment with the H_v inhibitor Zn^{2+}. **C)** SEM image of a *C. braarudii* cell treated with 2-GBI (15 µM) for 5 days showing the presence of many type-pH coccolith malformations. The inability of the coccoliths to interlock leads to the collapse of the

coccosphere during preparation for SEM imaging. Bar = 10 μm. **D)** Higher magnification SEM image of type-pH malformations induced by 15 μM 2-GBI. Bar = 5 μm.

Supplementary Tables

Table S1: Acclimation carbonate chemistry

Target pH		pH_{NBS}	H^+ (nmol kg^{-1})	pCO_2 (µatm)	CO_2 (µmol kg^{-1})	HCO_3^- (µmol kg^{-1})	CO_3^{2-} (µmol kg^{-1})	DIC (µmol kg^{-1})	TA (µmol kg^{-1})	$\Omega_{calcite}$
7.55	t_0	7.56 ± 0.02	37 ± 1	1608 ± 63	60 ± 2	1920 ± 11	40 ± 1	2020 ± 12	2058 ± 11	1.0 ± 0.0
	t_{fin}	7.63 ± 0.05	32 ± 3	1405 ± 174	52 ± 6	1931 ± 72	47 ± 2	2031 ± 77	2055 ± 67	1.1 ± 0.1
7.85	t_0	7.80 ± 0.02	21 ± 1	957 ± 51	36 ± 3	1987 ± 11	72 ± 2	2095 ± 12	2205 ± 12	1.8 ± 0.1
	t_{fin}	7.82	26	1231	46	2097	63	2206	2259	1.5
8.15	t_0	8.14 ± 0.01	10 ± 0	423 ± 9	16 ± 1	1928 ± 11	153 ± 5	2097 ± 16	2346 ± 23	3.6 ± 0.1
	t_{fin}	8.01 ± 0.03	13 ± 1	562 ± 41	21 ± 3	1882 ± 1	111 ± 9	2014 ± 6	2166 ± 20	2.6 ± 0.2
8.45	t_0	8.43 ± 0.01	5 ± 0	205 ± 2	8 ± 0	1825 ± 11	283 ± 6	2115 ± 17	2556 ± 24	6.7 ± 0.1
	t_{fin}	8.27 ± 0.03	7 ± 0	300 ± 21	11 ± 1	1819 ± 25	194 ± 12	2024 ± 25	2306 ± 34	4.6 ± 0.3
8.75	t_0	8.75 ± 0.01	2 ± 0	87 ± 1	3 ± 0	1596 ± 9	511 ± 10	2110 ± 19	2863 ± 31	12.2 ± 0.2
	t_{fin}	8.60 ± 0.01	3 ± 0	136 ± 2	5 ± 0	1781 ± 11	407 ± 11	2192 ± 20	2761 ± 33	9.7 ± 0.3

n=3 in all cases except pH 7.85 t_{fin} where n = 1 due to samples lost during analysis. t_0 = start of experiment, t_{fin} = end of experiment (5 d). pH_{NBS} = pH scale defined by the National Bureau of Standards (as opposed to pH_{total} scale); DIC = dissolved inorganic carbon; TA = total alkalinity, $\Omega_{calcite}$ = saturation state of calcite (>1 = indicates supersaturated)

Table S2: Comparison of treatments that induce coccolith malformations in *C. braarudii*

Treatment	Malformed coccoliths (% ± SE)	Type pH malformations (% ± SE)	Reference
**Cytoskeleton disruption**			
Control	18.0 ± 2.0	ND	(20)
Cytochalasin B (1.5 µM)	55.8 ± 2.6	ND	
Vinblastine (2 µM)	39.3 ± 0.1	ND	
**Nutrient limitation**			
Control (220 µM N, 9 µM P)	7.8 ± 1.0	ND	(21)
N-limited (20 µM)	22.1 ± 2.1	ND	
P-limited (1.5 µM)	31.6 ± 1.1	ND	
**Light**			
Control (20 µmol m^{-2} s^{-1})	3.9 ± 0.3	ND	(21)
High light (300 µmol m^{-2} s^{-1})	13.6 ± 3.1	ND	
**Germanium treatment**			
Control (5 µM Si)	20.2 ± 0.1	ND	(22)
0.1 µM Ge	23.9 ± 0.0	ND	
0.25 µM Ge	29.5 ± 0.1	ND	
0.5 µM Ge	65.9 ± 0.1	ND	
1 µM Ge	74.1 ± 0.1	ND	
**H^+ channel blocker**			
Control	3.5 ± 0.7	0.3 ± 0.2	This study
Zn (35 µM)	26.8 ± 1.0	13.6 ± 0.4	
2GBI (15 µM)	52.8 ± 6.2	20.6 ± 3.8	
**pH treatment**			
pH 8.75 (8.60)	3.3 ±2.1	0 ± 0	This study
pH 8.45 (8.27)	10.5 ±2.5	0 ± 0	
pH 8.15 (8.01)	42.1 ± 4.2	2.9 ± 1.1	
pH 7.85 (7.80)	70.0 ± 2.6	26.2 ± 2.0	
pH 7.55 (7.63)	81.0 ± 5.0	39.3 ± 6.8	
Values in parentheses indicate final pH			

ND = not detected. Malformations in this category were not observed in this study. Triplicate cultures were examined in all cases.

Table S3: Potential adaptive responses of *C. braarudii* following loss of H^+ channel function

Potential adaptation	Possible consequences
**1) Restore H^+ channel function**	
Reduce pH_{cyt}	Maintaining a lower pH_{cyt} will help to restore H^+ electrochemical gradient, but is likely to interfere with many aspects of metabolism
Increase Vm	Depolarisation of the resting V_m will help to restore the H^+ electrochemical gradient, but will have major consequences for many other aspects of membrane transport. H^+ efflux through H^+ channels may be feasible through repetitive transient depolarisations of V_m (action potentials), although slow activation kinetics of H_v channels are not ideally suited to rapid activation.
Modify H^+ channel gating	Adaptive shift of the activation potential to a more negative V_m will allow H^+ channels to operate at lower external pH. However, H^+ channel activity remains constrained by pmf, as outward H^+ electrochemical gradient is still required for H^+ efflux.
**2) Reduce H^+ load**	
Uptake of HCO_3^- rather than CO_2	Greater HCO_3^- uptake will result in increased consumption of H^+, but HCO_3^- transport is energetically more costly than CO_2 uptake. Cells would not benefit from elevated seawater CO_2.
Lower calcification rate	A lower calcification rate will reduce intracellular H^+ production, but may prevent formation of a complete coccosphere, or interfere with other aspects of coccolith function (e.g. reduce ability of *C. braarudii* placoliths to interlock) that are critical for ecological success.
**3) Use alternative mechanisms for H^+ efflux**	
Use energised H^+ transport	Use of energised mechanisms of H^+ transport (e.g. Na^+/H^+ exchange or H^+-ATPase) will allow H^+ efflux against an unfavourable H^+ electrochemical gradient, although energised H^+ transport is unlikely to match the capacity for rapid H^+ efflux that is required in heavily calcified species and would have increased energetic costs.

Table S4. External and pipette solution compositions for patch clamp recording of *C. braaudii*.

	C. braarudii	
	**External solution (mM)**	**Internal solution (mM)**
**NaCl**	450	
**KCl**	8	
**$CaCl_2$**	10	
**$MgCl_2$**	30	5
**$MgSO_4$**	16	
**$NaHCO_3$**	2	
**HEPES**	20	100
**K-glutamate**		200
**EGTA**		5
**pH**	8.15	7.5

Supplementary text

The electrical charge across the plasma membrane of eukaryote cells is known as the membrane potential and plays an important role in the movement of ions into and out of the cell. In most eukaryotic cells, efflux of K^+ through the activity of voltage-gated K^+ channels is central to maintaining the membrane potential. In coccolithophores the major outward current at positive membrane potentials is not K^+, but H^+. The membrane physiology of coccolithophores is therefore dominated by H^+ fluxes, representing the highly specialised nature of pH homeostasis within these organisms.

Movement of H^+ through H^+ channels is not energised and is therefore entirely dependent on the transmembrane electrochemical gradient for H^+, which is determined by the combination of the membrane potential (i.e. the electrical charge across the membrane) and the concentration gradient for H^+ across the membrane. H^+ conductance through the H^+ channel can only occur down the electrochemical gradient. H^+ channels therefore differ from other forms of H^+ transport, such as H^+ pumps (H^+-ATPases), which can utilise energy from the hydrolysis of ATP to transport H^+ against the electrochemical H^+ gradient.

H^+ channel activity is controlled by channel gating, whereby the channel exists in open or closed states. The H_v class of H^+ channels are voltage-gated and become activated following membrane depolarisation (i.e. the channel opens when membrane potential becomes less negative). Patch clamp experiments allow the membrane potential and internal and external ionic conditions to be manipulated and corresponding currents measured. In coccolithophores, the activation potential of the H^+ current is finely poised at a potential slightly more positive than the resting membrane potential. This means that even a small depolarisation of the membrane will activate the channel, causing it to switch to the open configuration to allow H^+ to flow down the electrochemical gradient out of the cell (15). The rapid H^+ efflux decreases the intracellular concentration of H^+, increasing intracellular pH. Moreover, the movement of H^+ out of the cell hyperpolarises the plasma membrane (makes the membrane potential more negative). Both of these effects reduce the transmembrane electrochemical H^+ gradient and act to deactivate the H^+ channel, providing a direct feedback loop to control H^+ efflux.

A particular feature of H_v channels is that they exhibit pH-dependence of voltage-gating (Figure S3) (23). Lowering of cytoplasmic pH (an increase intracellular H^+) not only increases the transmembrane electrochemical gradient for H^+, but it also causes the activation potential of the H^+ channel to shift to a more negative value. As the activation potential is already close to the resting membrane potential, shifting the activation potential to a more negative value results in channel activation. The resultant H^+ efflux acts to raise cytoplasmic pH, which returns the activation potential of the H^+ channel to a more positive value, deactivating the channel. Thus, the H^+ channel is activated when cytoplasmic pH decreases and closes as soon as cytoplasmic pH is restored, allowing the cell to finely control cytoplasmic pH. The degree of pH-dependent voltage-gating exhibited by H_v channels means that the direction of H^+ transport through these channels is nearly always outward.

Although these properties would appear to make H^+ channel mediated pH homeostasis an incredibly effective mechanism of pH homeostasis, one consequence of pH-dependent voltage-gating is sensitivity to changes in extracellular pH. Lowering extracellular pH reduces the outward electrochemical H^+ gradient, but it also shifts the activation potential of the H^+ channel to a more positive value. Thus, the activation potential of H^+ channel becomes much more positive than resting membrane potential and activation of the H^+ channel would therefore require a much more substantial cytoplasmic acidification.

The reliance on H^+ channels for pH homeostasis means coccolithophores are likely to be particularly sensitive to changes in extracellular pH. One way to overcome this sensitivity would be for the cell to adjust its resting membrane potential following changes in extracellular pH, so that H^+ channel activation is once again finely poised. However, as the resting membrane potential influences all other aspects of membrane transport, there will be limits to the extent to which resting membrane potential can change and any adjustments may have much wider consequences for cell physiology.

References

1. von Dassow P, *et al.* (2018) Over-calcified forms of the coccolithophore *Emiliania huxleyi* in high-CO_2 waters are not preadapted to ocean acidification. *Biogeosciences* 15(5):1515-1534.
2. Bach LT, Riebesell U, Gutowska MA, Federwisch L, & Schulz KG (2015) A unifying concept of coccolithophore sensitivity to changing carbonate chemistry embedded in an ecological framework. *Prog Oceanogr* 135:125-138.
3. Bretherton L, *et al.* (2019) Day length as a key factor moderating the response of coccolithophore growth to elevated pCO_2. *Limnology and Oceanography* 64(3):1284-1296.
4. Fiorini S, Middelburg JJ, & Gattuso JP (2011) Effects of elevated CO_2 partial pressure and temperature on the coccolithophore *Syracosphaera pulchra*. *Aquatic Microbial Ecology* 64(3):221-232.
5. Gafar NA, Eyre BD, & Schulz KG (2019) Particulate inorganic to organic carbon production as a predictor for coccolithophorid sensitivity to ongoing ocean acidification. *Limnol Oceanogr Lett* 4(3):62-70.
6. Krug SA, Schulz KG, & Riebesell U (2011) Effects of changes in carbonate chemistry speciation on *Coccolithus braarudii*: a discussion of coccolithophorid sensitivities. *Biogeosciences* 8(3):771-777.
7. Langer G, *et al.* (2006) Species-specific responses of calcifying algae to changing seawater carbonate chemistry. *Geochem Geophy Geosy* 7.
8. Langer G, Nehrke G, Probert I, Ly J, & Ziveri P (2009) Strain-specific responses of *Emiliania huxleyi* to changing seawater carbonate chemistry. *Biogeosciences* 6(11):2637-2646.
9. Liu YW, Eagle RA, Aciego SM, Gilmore RE, & Ries JB (2018) A coastal coccolithophore maintains pH homeostasis and switches carbon sources in response to ocean acidification. *Nature communications* 9: 2857.
10. Sett S, *et al.* (2014) Temperature modulates coccolithophorid sensitivity of growth, photosynthesis and calcification to increasing seawater pCO_2. *Plos One* 9(2).
11. Supraha L, Gerecht AC, Probert I, & Henderiks J (2015) Eco-physiological adaptation shapes the response of calcifying algae to nutrient limitation. *Scientific reports* 5: 16499.
12. Zondervan I, Rost B, & Riebesell U (2002) Effect of CO_2 concentration on the PIC/POC ratio in the coccolithophore *Emiliania huxleyi* grown under light-limiting conditions and different daylengths. *J Exp Mar Biol Ecol* 272(1):55-70.
13. White MM, *et al.* (2018) Calcification of an estuarine coccolithophore increases with ocean acidification when subjected to diurnally fluctuating carbonate chemistry. *Mar Ecol Prog Ser* 601:59-76.
14. Blanco-Ameijeiras S, *et al.* (2016) Phenotypic variability in the coccolithophore *Emiliania huxleyi*. *Plos One* 11(6) 0157697.
15. Taylor AR, Chrachri A, Wheeler G, Goddard H, & Brownlee C (2011) A voltage-gated H^+ channel underlying pH homeostasis in calcifying coccolithophores. *PLoS Biol* 9(6):e1001085.

16. Taylor AR, Brownlee C, & Wheeler GL (2012) Proton channels in algae: reasons to be excited. *Trends Plant Sci* 17(11):675-684.
17. Taylor AR & Brownlee C (2003) A novel Cl^- inward-rectifying current in the plasma membrane of the calcifying marine phytoplankton *Coccolithus pelagicus*. *Plant Physiology* 131(3):1391-1400.
18. Helliwell KE, *et al.* (2020) A novel single-domain Na^+-selective voltage-gated channel in photosynthetic eukaryotes. *Plant Physiol* 184(4):1674-1683.
19. Hong L, Kim IH, & Tombola F (2014) Molecular determinants of Hv1 proton channel inhibition by guanidine derivatives. *Proc Natl Acad Sci U S A* 111(27):9971-9976.
20. Langer G, *et al.* (2022) The effect of cytoskeleton inhibitors on coccolith morphology in *Coccolithus braarudii* and *Scyphosphaera apsteinii*. *bioRxiv*:2022.2002.2018.480830.
21. Langer G, *et al.* (2022) Distinct physiological responses of *Coccolithus braarudii* life cycle phases to light intensity and nutrient availability. *bioRxiv*:2022.2002.2017.480838.
22. Langer G, *et al.* (2021) Role of silicon in the development of complex crystal shapes in coccolithophores. *New Phytol*. 231(5): 1845-1857.
23. DeCoursey TE (2013) Voltage-gated proton channels: molecular biology, physiology, and pathophysiology of the H(V) family. *Physiol Rev* 93(2):599-652.
